# Development and clinical validation of CT-based regional modified Centiloid method for amyloid PET

**DOI:** 10.1186/s13195-022-01099-0

**Published:** 2022-10-20

**Authors:** Soo-Jong Kim, Hongki Ham, Yu Hyun Park, Yeong Sim Choe, Young Ju Kim, Hyemin Jang, Duk L. Na, Hee Jin Kim, Seung Hwan Moon, Sang Won Seo

**Affiliations:** 1 Department of Neurology, Samsung Medical Center, Sungkyunkwan University School of Medicine, 81 Irwon-ro, Gangnam-gu, Seoul, 06351 Republic of Korea; 2 Neuroscience Center, Samsung Medical Center, Seoul, Republic of Korea; 3 Department of Health Sciences and Technology, SAIHST, Sungkyunkwan University, Seoul, Republic of Korea; 4 Department of Intelligent Precision Healthcare Convergence, Sungkyunkwan University, Suwon, Republic of Korea; 5 Department of Digital Health, SAIHST, Sungkyunkwan University, Seoul, Republic of Korea; 6 Stem Cell and Regenerative Medicine Institute, Samsung Medical Center, Seoul, Republic of Korea; 7 Department of Nuclear Medicine, Samsung Medical Center, Sungkyunkwan University School of Medicine, Seoul, Republic of Korea; 8 Alzheimer’s Disease Convergence Research Center, Samsung Medical Center, Seoul, Republic of Korea

**Keywords:** Amyloid PET, CT, Regional modified Centiloid, Clinical validation

## Abstract

**Background:**

The standard Centiloid (CL) method was proposed to harmonize and quantify global ^18^F-labeled amyloid beta (Aβ) PET ligands using MRI as an anatomical reference. However, there is need for harmonizing and quantifying regional Aβ uptakes between ligands using CT as an anatomical reference. In the present study, we developed and validated a CT-based regional direct comparison of ^18^F-florbetaben (FBB) and ^18^F-flutemetamol (FMM) Centiloid (rdcCL).

**Methods:**

For development of MRI-based or CT-based rdcCLs, the cohort consisted of 63 subjects (20 young controls (YC) and 18 old controls (OC), and 25 participants with Alzheimer’s disease dementia (ADD)). We performed a direct comparison of the FMM-FBB rdcCL method using MRI and CT images to define a common target region and the six regional VOIs of frontal, temporal, parietal, posterior cingulate, occipital, and striatal regions. Global and regional rdcCL scales were compared between MRI-based and CT-based methods. For clinical validation, the cohort consisted of 2245 subjects (627 CN, 933 MCI, and 685 ADD).

**Results:**

Both MRI-based and CT-based rdcCL scales showed that FMM and FBB were highly correlated with each other, globally and regionally (*R*^2^ = 0.96~0.99). Both FMM and FBB showed that CT-based rdcCL scales were highly correlated with MRI-based rdcCL scales (*R*^2^ = 0.97~0.99). Regarding the absolute difference of rdcCLs between FMM and FBB, the CT-based method was not different from the MRI-based method, globally or regionally (*p* value = 0.07~0.95). In our clinical validation study, the global negative group showed that the regional positive subgroup had worse neuropsychological performance than the regional negative subgroup (*p* < 0.05). The global positive group also showed that the striatal positive subgroup had worse neuropsychological performance than the striatal negative subgroup (*p* < 0.05).

**Conclusions:**

Our findings suggest that it is feasible to convert regional FMM or FBB rdcSUVR values into rdcCL scales without additional MRI scans. This allows a more easily accessible method for researchers that can be applicable to a variety of different conditions.

**Supplementary Information:**

The online version contains supplementary material available at 10.1186/s13195-022-01099-0.

## Background

The standard Centiloid (CL) method was recently proposed to harmonize and quantify ^18^F-labeled amyloid beta (Aβ) PET ligands using ^11^C-labeled Pittsburgh compound B (^11^C-PiB) images as a reference [1–5]. The equations derived for conversion of SUVR into CL scales in previous studies used ^11^C-PiB PET and ^18^F-labeled amyloid PET images and applied the equations to ^18^F-labeled Aβ ligands to convert standard CL scales using the ^11^C-PiB ligand [1–5]. However, ^11^C-PiB PET ligands are not available in most medical centers due to the limitations described above. Therefore, in our previous study, a direct comparison CL (dcCL) method that harmonizes ^18^F-florbetaben (FBB) and ^18^F-flutemetamol (FMM) PET ligands without ^11^C-PiB images was developed [6]. Our previous study suggested that standard CL and dcCL were highly correlated, but the variation between FBB and FMM was smaller with the dcCL method than with the standard CL method [6].

Because PET has low spatial resolution, MRI can be used as an anatomical reference to quantify PET uptakes. Therefore, both the standard CL process and the dcCL method require PET and MR images to measure CL scales. However, in practical assessments, MRI scans can involve risk for certain patients who have devices such as cardiac pacemakers or implantable cardioverter defibrillators (ICDs) [7], and both PET and MRI scans can be a financial burden. Because PET and CT images can be acquired through only one scan with a PET-CT scanner, CT images can be used as anatomical reference images to PET images instead of MRI. There could be a difference between MRI-based regional dcCL (rdcCL) and CT-based rdcCL if coregistering PET to MRI differs to coregistering it to CT (which should be very close to the same head orientation since it was acquired before PET scan) and if warping the MRI to the template produces a vastly non-linear transformation in comparison to warping the CT to the template.

Both the standard CL and the dcCL methods generate global CL scales but not regional brain Aβ uptake information. However, in our previous study, regionally increased Aβ uptake was shown to be associated with cognitive impairment [8]. In particular, patients with striatal involvement of Aβ showed worse prognosis [9]. Therefore, it is important to harmonize Aβ uptake between Aβ ligands globally and regionally for earlier detection and better prediction of prognosis.

In the present study, CT-based rdcCL scales were developed using a head-to-head comparison cohort of participants who underwent FMM and FBB. Because our MRI-based global dcCL method previously showed the two ligands to be mutually highly correlated, we hypothesized that MRI- or CT-based rdcCL scales of FMM would correlate with those of FBB. In addition, hypothetically, our CT-based rdcCL scales are comparable to MRI-based rdcCL scales regarding reliability and precision. Finally, to validate the clinical efficacy of the newly developed CT-based rdcCL scales, the effects of regionally increased Aβ uptake on cognitive impairment, including striatal involvement of Aβ, were explored. We hypothesized differences in verbal memory test score between the G(−)R(−) and the G(−)R(+) groups because the test might reflect early changes along the AD continuum. We also hypothesized differences in global cognition score between the G(+)Str(−) and the G(+)Str(+) groups because G(+) groups might be included in the late stage of the AD continuum.

## Materials and methods

### Participants

To develop MRI- or CT-based rdcCLs, the study cohort included 63 subjects: 20 young controls (YCs), 18 old controls (OCs), and 25 individuals with Alzheimer’s disease dementia (ADD). The subjects underwent paired FMM and FBB PET-CT and three-dimensional (3D) T1 MRI. Healthy YCs were younger than 40 years and had normal cognitive function and no history of neurological or psychiatric disorders. The OCs were older than 65 years and had normal cognitive function determined using neuropsychological tests and no history of neurological or psychiatric disorders. Participants diagnosed with MCI had to meet Petersen’s criteria [10] and show objective memory impairment one standard deviation (SD) below the norm in at least one memory test. ADD was diagnosed based on the National Institute on Aging-Alzheimer’s Association (NIA-AA) research criteria for probable AD [11]. The FMM and FBB PET scans as well as CT and MRI scans in 18 OCs and 25 ADDs were used to create the target VOIs, and the scans in 20 YCs and 25 ADDs were used to create the 0 and 100 anchor points.

All participants underwent clinical interviews, neurological and neuropsychological examinations, and laboratory tests including complete blood count, blood chemistry, thyroid function tests, syphilis serology, and vitamin B12/folate levels. The absence of structural lesions including cerebral infarctions, brain tumors, vascular malformations, and hippocampal sclerosis was confirmed based on brain MRI.

The Institutional Review Board of Samsung Medical Center approved the study protocol, and all methods were performed according to the approved guidelines. Written consent was obtained from each participant.

### MRI data acquisition

Standardized 3D T1 turbo field echo images were acquired from all participants at Samsung Medical Center using the same 3.0 T MRI scanner (Philips Achieva; Philips Healthcare, Andover, MA, USA). The detailed parameters are described in Additional file 1: Supplementary Methods 1.

### Aβ PET-CT data acquisition

Participants underwent FMM and FBB PET at Samsung Medical Center using a Discovery STe PET/CT scanner (GE Medical Systems, Milwaukee, WI, USA) in 3D scanning mode that examined 47 slices 3.3-mm in thickness spanning the entire brain [12, 13]. Paired FMM and FBB PET images were acquired on two separate days; the mean interval time (4.0 ± 2.5 months across all groups) was not different among the three groups (*p* = 0.89). Among the 63 images in the head-to-head dataset, FMM PET was performed first in half of the participants (total 36: 19 ADD, 6 OCs, and 11 YCs) and FBB PET first in the other half of the participants (total 27: 6 ADD, 12 OCs, and 9 YCs). According to the protocols for the ligands proposed by the manufacturers, a 20-min emission PET scan with dynamic mode (consisting of 4 × 5 min frames) was performed 90 min after injection of a mean dose of 185 MBq of FMM or 311.5 MBq of FBB. 3D PET images were reconstructed in a 128 × 128 × 47 matrix with a voxel size of 2 mm × 2 mm × 3.27 mm using the ordered-subsets expectation maximization algorithm (FMM iterations = 4 and subset = 20; FBB iterations = 4 and subset = 20).

CT images were acquired using a 16-slice helical CT (140 KeV, 80 mA; 3.75-mm section width) for attenuation correction and were reconstructed in a 512 × 512 matrix with a voxel size of 0.5 mm × 0.5 mm × 3.27 mm. The kernel (i.e., smoothing filter) size of 5 mm was used for FMM according to the vendor’s recommendation. However, since there was no recommendation of smoothing for FBB, we did not do perform it.

### Regional visual assessment and rdcCL scales

Two experienced neurologists visually quantified FMM and FBB images [14] in our head-to-head dataset. Inter-rater agreement was excellent for FMM (Fleiss *k* = 0.86–0.97) and FBB (Fleiss *k* = 0.9–1.0) for five regions. For discordant cases, another experienced nuclear medicine doctor was consulted. After individual ratings were performed, the final visual positivity was determined based on the majority agreement regarding visual reading results.

### Development of MRI-based global and regional CTX VOIs

**Fig. 1:**
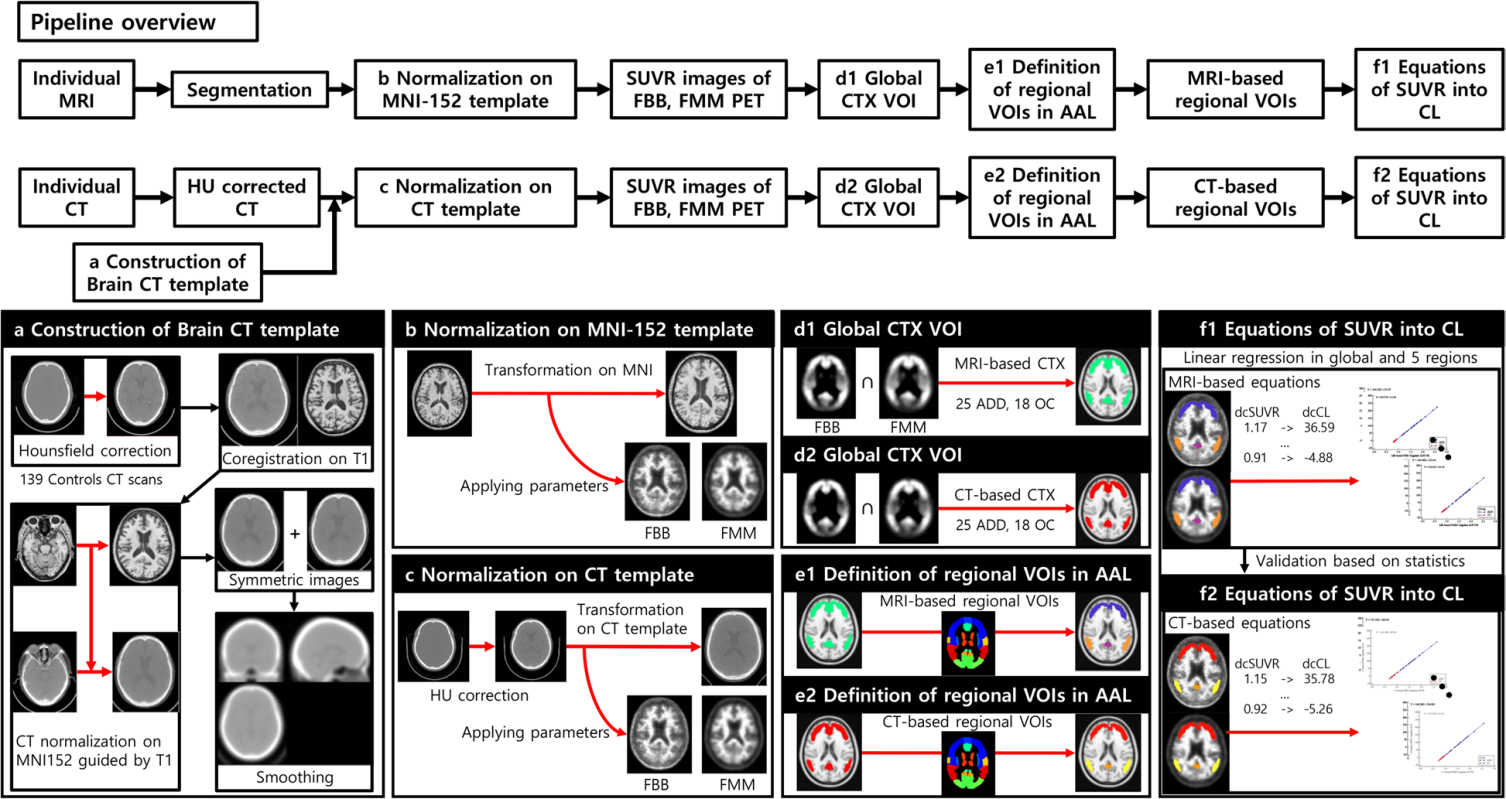
Overview of the processing pipeline for regional Centiloid in CT-based and MRI-based methods. a Construction of a brain CT template. b Normalization of MRI and PET images onto the MNI-152 template. c Normalization of HU-corrected CT and PET images on a generated CT template. d1 The MRI-based method created global CTX VOIs for target regions in common for FMM and FBB PET. d2 The CT-based method created global CTX VOIs for target regions common in FMM and FBB PET. e1 Definition of MRI-based regional VOIs in AAL. e2 Definition of CT-based regional VOIs in AAL. f1 Equations for direct conversion of MRI-based rdcSUVRs into rdcCLs globally and in six regions (frontal, PC, parietal, striatum, occipital, and temporal). f2 Equations for direct conversion of CT-based rdcSUVRs into rdcCLs globally and in six regions

The method overview is shown in Fig. 1. To develop MRI-based rdcCLs, we followed the CL process for preprocessing (i.e., creating SUVR parametric PET images on MNI space) to MRI, FMM, and FBB PET images as described in Klunk et al. The processing details are provided in the original CL manuscript [3]. Briefly, individual MR images were co-registered onto the MNI-152 template, and then individual PET images were co-registered onto the corresponding MRI images. The PET and MRI images were spatially normalized (Fig. 1b) using transformation parameters of the SPM8 unified segmentation method of T1-weighted MRIs. The whole cerebellum (WC) mask was used as the reference region from the GAAIN website and SUVR parametric PET images of FMM and FBB using the WC mask created and used for global and rdcCLs. To define the common cortical target region with amyloid accumulation distributed for FMM and FBB PET, 25 Aβ PET-positive (+) ADD participants and 18 Aβ PET-negative (−) OCs were included for all PET ligands in a head-to-head cohort [6]. The method in the original publication was used to create a common cortical target VOI (FMM-FBB global CTX VOI) for both FMM and FBB PET, and the details are described in the original publication (Fig. 1d1) [6]. Individual rdcSUVR values in the FMM-FBB global CTX VOI were calculated in all PET images. MRI-based regional VOIs were defined by overlapping MRI-based FMM-FBB global CTX VOI and the AAL atlas (Fig. 1e1) [15].

We selected the common target areas as VOIs and divided them into six regions. The regional VOIs were named the frontal, lateral temporal, occipital, parietal, posterior cingulate, and striatal areas. The frontal VOI comprised parts of the superior and middle frontal gyri; medial part of the superior frontal gyrus; opercular part of the inferior frontal gyrus; triangular part of the inferior frontal gyrus; supplementary motor area; orbital parts of the superior, middle, and inferior orbital frontal gyri. The lateral temporal VOI was composed of parts of the superior, middle, and inferior temporal gyri. The occipital VOI involved parts of the calcarine, cuneus, superior, middle, and inferior occipital gyri. The parietal VOI was parts of superior and inferior parietal, supramarginal and angular gyri, and precuneus). The posterior cingulate VOI comprises parts of the posterior cingulate gyri, and the striatal VOI is parts of the caudate and putamen. rdcSUVR values were calculated using the six regional VOIs.

### Development of CT-based global and regional CTX VOIs

For constructing the brain CT template, 139 CT scans were collected from normal controls (NCs) in another dataset who underwent FBB-PET CT. In the PET-CT scanner, the CT scan was low dose to reduce patient exposure to radiation. The brain CT template was created using corrected Hounsfield units (HU) of brain tissues in the CT images. The details of the HU correction approach are described in the original methodology paper [16]. Briefly, as shown in Fig. 1a, total CT images were reoriented. Intensities of the images were scaled to boost HU of brain tissues. The HU-corrected CT images were co-registered onto corresponding T1 MR images. Individual T1 MR images were spatially normalized on MNI space, and spatial normalization parameters of T1 MR images were applied to corresponding HU-corrected CT images. The normalized CT images were flipped to create a symmetric template, and the mean image was created using the normalized CT images. Gaussian smoothing at 8 mm was applied to the template to remove noise.

HU correction processing was performed on individual CT images. FMM and FBB PET images were co-registered onto corresponding HU-corrected CT images, and the PET images were spatially normalized using parameters of each HU-corrected CT image onto MNI space using the created brain CT template (Fig. 1c). Using the normalized FMM and FBB PET images with WC mask as the reference region, CT-based SUVR parametric PET images were created. These images were used to generate an FMM-FBB global CTX VOI in the same manner as described for the MRI-based FMM-FBB method (Fig. 1d2). In addition, individual rdcSUVR values were calculated using the CT-based FMM-FBB global CTX VOIs. CT-based regional VOIs were also defined by overlapping CT-based FMM-FBB global CTX VOI and the AAL atlas (Fig. 1e2). The sub-regions of AAL in the CT-based global CTX VOI were merged into six regions (frontal, PC, parietal, striatum, occipital, and temporal), which were used to calculate rdcSUVR values.

### Development of MRI- and CT-based rdcCL

The dcCL method was used to derive equations from rdcSUVR values using created FMM-FBB VOIs for direct conversion [6]. Figures 1f1 and f2 show the summary of methods; each method shows the regression equations derived from rdcSUVR and rdcCL of MRI-based and CT-based methods globally and in six regions.

The FMM-FBB VOIs of the CT-based method were applied to FMM and FBB PET to acquire rdcSUVRs, and FMM-FBB VOIs of the MRI-based method were used to validate the CT-based method. First, the equations of rdcCL conversion from MRI- and CT-based rdcSUVR and rdcCL scales were derived using the CL formula globally and in six regions. Second, rdcCL scales of MRI- and CT-based methods were calculated using the rdcCL conversion equations globally and for the six regions.

### Validation of the clinical efficacy of CT-based rdcCLs in the independent cohort

To validate the clinical efficacy of CT-based rdcCLs, 2245 FMM (*n*=1203) and FBB (*n*=1042) PET scans in ADD, aMCI, and cognitive normal (CN) groups were analyzed. All participants with aMCI met the following criteria: (1) subjective memory complaints by participants or caregiver; (2) objective memory dysfunction, as evidenced by low scores from evaluations on verbal or visual memory tests (*z*-score < −1.0); (3) no significant impairment in activities of daily living; and (4) non-demented. All participants underwent FMM or FBB PET at Samsung Medical Center using Discovery STe PET/CT scanners (GE Medical Systems, Milwaukee, WI, USA). We performed receiver operating characteristic (ROC) analysis to determine rdcCL cutoff values in a head-to-head comparison dataset of 29 AD dementia, 27 aMCI, and 27 OC subjects. We used FBB rdcCL scales to determine rdcCL cutoff values. Visual assessment scores were used as standard of truth in global and regional areas. The cutoff values were 30.52, 17.14, 33.21, 25.54, 50.64, and 40.1 in global, frontal, parietal, temporal, posterior cingulate, and striatal regions, respectively.

The group was classified into four groups based on global and striatal rdcCL cutoffs. First, based on global Aβ rdcCL scales, the cohort was classified as global (−) and global (+). The global (−) group was further classified into regional (−) and regional (+) groups based on regional cutoffs for at least one region. In addition, the global (+) group was further classified into striatal (−) and striatal (+) groups based on striatal cutoffs. Thus, the cohort was classified into four groups: global (−) and regional (−) Aβ: G(−)R(−); global (−) and regional (+) Aβ: G(−)R(+); global (+) and striatal (−) Aβ: G(+)Str(−); and global (+) and striatal (+) Aβ: G(+)Str(+).

All participants underwent neuropsychological testing using the Seoul Neuropsychological Screening Battery 2nd edition (SNSB-II) including the Seoul Verbal Learning Test (SVLT) delayed recall and Clinical Dementia Rating Scale Sum of Boxes (CDR-SOB) [17, 18]. The detailed items are described in Additional file 1: Supplementary Methods 2.

### Statistical analysis

In the head-to-head cohort, group difference and ROC analysis were performed between regional visual positivity and MRI- and CT-based rdcCL scales in order to validate rdcCL scales. Regression analysis was performed for reliability between FMM and FBB PET ligands or between MRI-based and CT-based methods using rdcSUVR and rdcCL scales globally and regionally. Regression was also performed to derive global and rdcCL formulas from the head-to-head cohort. In order to convert rdcSUVR to rdcCL scales, we used data from 25 ADD and 20 YC who underwent both FMM and FBB PET. Briefly, the method directly converted the rdcSUVR values of the FMM-FBB CTX VOI into rdcCL scales using the CL conversion equation.

$$\textrm{CL}=100\times \left({\textrm{SUVR}}_{\textrm{ind}}-{\textrm{SUVR}}_{\textrm{YC}-0}\right)\kern0.5em /\left({\textrm{SUVR}}_{\textrm{ADD}-100}-{\textrm{SUVR}}_{\textrm{YC}-0}\right)$$

where SUVR_ind_ represents the individual SUVR values of all YC-0 and ADD-100 participants, and SUVR_YC-0_ and SUVR_ADD-100_ represent respective group mean SUVR values. The CL equation was derived separately for FMM and FBB PET and applied to the FMM and FBB SUVR, respectively, from the FMM-FBB rdcCL VOIs. This SUVR from FMM-FBB rdcCL VOIs was defined as rdcSUVR to make rdcCL equations which convert rdcSUVR into rdcCL scales.

In order to calculate the correlation between rdcCL MRI-based and rdcCL CT-based, we used data from 25 ADD and 20 YC who underwent both 3D MRI and PET-CT scan.

For precision, the differences in rdcCL scales between FMM and FBB ligands or between MRI-based and CT-based methods were investigated using Bland-Altman plots [19]. The absolute value differences between rdcCL scales of MRI-based and CT-based methods or between rdcCL scales derived based on FMM and FBB ligands were compared using a generalized estimating equation (GEE).

In an independent cohort for clinical validation, the chi-square test for categorical variables and analysis of covariance (ANCOVA) for continuous variables were used to compare the demographics and frequency of APOE4 genotype and MMSE scores among the four groups. To investigate the neuropsychological results among the four groups, ANCOVA was performed after controlling for age and apolipoprotein E ε4 (*APOE4-ε4*) carrier.

SPSS version 24.0 (SPSS Inc., Chicago, IL, USA) was used for GEE and MedCalc Statistical Software version 17.9.2 (Ostend, Belgium; 2017) for correlation, linear regression, ANCOVA, and Bland-Altman analyses.

## Results

### Demographics of the participants

Table 1 shows the demographic information of the participants in the rdcCL development cohort. The average age (SD) of all 63 participants was 58.7 (19.4) years, and 58.7% were females. The frequency of *APOE*-*ε4* carriers was 39.7%. The average scores (SD) of the Seoul Verbal Learning Test delayed recall (SVLT delayed recall), Mini-Mental State Examination (MMSE), and Clinical Dementia Rating Scale Sum of Boxes (CDR SOB) were 3.2 (3.4), 26.6 (5.5), and 3.4 (3.9), respectively. Table 2 shows participant demographics and clinical findings of subgroups for rdcCL validation.

**Table 1:** Participant demographics and clinical findings in head-to-head and clinical validation cohorts

	Head-to-head cohort	Clinical validation cohort		
		FMM	FBB	*p*-value
N	63	1203	1042	
Age (years)	58.7±19.4	69.7±9.8	69.4±10.8	0.63
Gender (F), *N* (%)	37 (58.7)	671 (55.8)	589 (56.5)	0.72
APOE4 carriers, *N* (%)	25 (39.7)	417 (35.4)	426 (42.7)	0.001
SVLT delayed recall, mean ± SD	3.2±3.4	3.3±3.2	2.7±3.1	<0.001
MMSE, mean ± SD	26.6±5.5	24.9±4.9	24±5.5	<0.001
CDR-SOB, mean ± SD	3.4±3.9	2.4±2.8	3.2±3.3	<0.001
NC/aMCI/ADD, *N*	38/0/25	346/554/303	281/379/382	

*ADD* Alzheimer’s disease dementia, *aMCI* amnestic mild cognitive impairment, *APOE-ε4* apolipoprotein E ε4 allele, *CDR-SOB* Clinical Dementia Rating Scale Sum of Boxes, *CN* cognitive normal, *F* female, *FBB*^18^F-florbetaben, *FMM*^18^F-flutemetamol, *MMSE* Mini-Mental State Examination, *aMCI* amnestic mild cognitive impairment, *SVLT* Seoul Verbal Learning Test

**Table 2:** Participant demographics and clinical findings of the subgroups

	G(−)R(−)	G(−)R(+)	G(+)Str(−)	G(+)Str(+)
N	815	243	85	1102
Age (years)	68.1±11.7	70.7±9.4	73.7±7.2	70.1±9.3
Gender (F) (*N*, %)	437 (53.6)	128 (52.7)	60 (70.6)	635 (57.6)
APOE4 carriers (*N*, %)	110 (14.1)	69 (29.4)	28 (34.6)	636 (59.1)
SVLT delayed recall, mean ± SD	5±3.2	3.8±3.1	3.4±3.4	1.4±2.2
MMSE, mean ± SD	27±3.5	25.9±4.5	25.4±4.5	22.3±5.5
CDR-SOB, mean ± SD	1.5±2	2±3	2.4±3	3.9±3.2
NC/aMCI/ADD, *N*	414/318/83	95/115/33	26/38/21	92/462/548
FBB/FMM, *N*	331/484	95/148	41/44	575/527

*ADD* Alzheimer’s disease dementia, *aMCI* amnestic mild cognitive impairment, *APOE-ε4* apolipoprotein E ε4 allele, *CDR-SOB* Clinical Dementia Rating Scale Sum of Boxes, *CN* cognitive normal, *F* female, *FBB*^18^F-florbetaben, *FMM*^18^F-flutemetamol, *G(−)R(−)*global (−) and regional (−) Aβ rdcCL scales, *G(−)R(+)*global (−) and regional (+) Aβ rdcCL scales, *G(+)Str(−)*global (+) and striatal (−) Aβ rdcCL scales, *G(+)Str(+)*global (+) and striatal (+) Aβ rdcCL scales, *MMSE* Mini-Mental State Examination, *aMCI* amnestic mild cognitive impairment, *SVLT* Seoul Verbal Learning Test

### Visual assessment and rdcCL scales

Most regional MRI- and CT-based rdcCL scales showed area under the curve (AUC) values higher than 0.9 for five regions classified by visual read as in Table 3. The five regions are significantly different for FMM and FBB PET (*p* < 0.001, Additional file 1: Figure S1).

**Table 3:** Visual assessment and rdcCL scales

		MRI-rdcCL			CT-rdcCL		
		AUC	Sensitivity (%)	Specificity (%)	AUC	Sensitivity (%)	Specificity (%)
FMM	Frontal	0.99	100	97.5	0.97	91.3	97.5
	PC	0.99	100	97.5	0.99	100	95
	Parietal	0.99	100	97.44	0.98	95.83	97.44
	Striatum	0.98	100	97.67	0.98	100	95.35
	Temporal	0.99	100	94.87	0.99	100	97.44
FBB	Frontal	0.98	88.89	100	0.95	85.19	94.44
	PC	0.99	100	90.7	0.99	100	95.35
	Parietal	1	100	100	0.99	100	97.37
	Striatum	0.96	91.67	97.44	0.97	91.67	97.44
	Temporal	0.99	96.55	97.06	0.99	93.1	97.06

Abbreviations: *FMM*^18^F-flutemetamol, *FBB*^18^F-florbetaben, *AUC* area under the curve, *rdcCL* Centiloid scales of FMM-FBB CTX VOI and regional VOIs, *PC* posterior cingulate

### CT-based rdcCLs

The spatial distribution of Aβ deposition in the CT-based CTX VOIs did not differ between FMM and FBB as shown in Additional file 1: Fig. S2d and 2e. The CT-based CTX VOI regions (Additional file 1: Fig. S2f) generally overlapped with MRI-based CTX VOI regions (Additional file 1: Fig. S2c). In total, 27,912 voxels were included in both VOIs for MRI-based and CT-based rdcCL, 4795 voxels were included in the only-MRI-based rdcCL, and 6275 voxels were included in the only-CT-based rdcCL (Additional file 1: Fig. S2g). The FMM and FBB SUVR values of the head-to-head cohort showed excellent linear correlation, and all *R*^2^ values in global and six regional VOIs were 0.97 (Additional file 1: Fig. S3b).

The regression equations to convert the FMM rdcSUVR into FMM rdcCL (Additional file 1: Fig. S4c) and FBB rdcSUVR into FBB rdcCL (Additional file 1: Fig. S4d) globally and regionally were calculated. The rdcCL scales between FMM and FBB were highly correlated globally and regionally (*R*^2^ = 0.97; Additional file 1: Fig. S5b) using the direct comparison of the CT-based method.

The results of MRI-based rdcCLs are described in Additional file 1: Supplementary Method 3.

### Reliability and precision in the MRI-based and CT-based rdcCLs

**Fig. 2:**
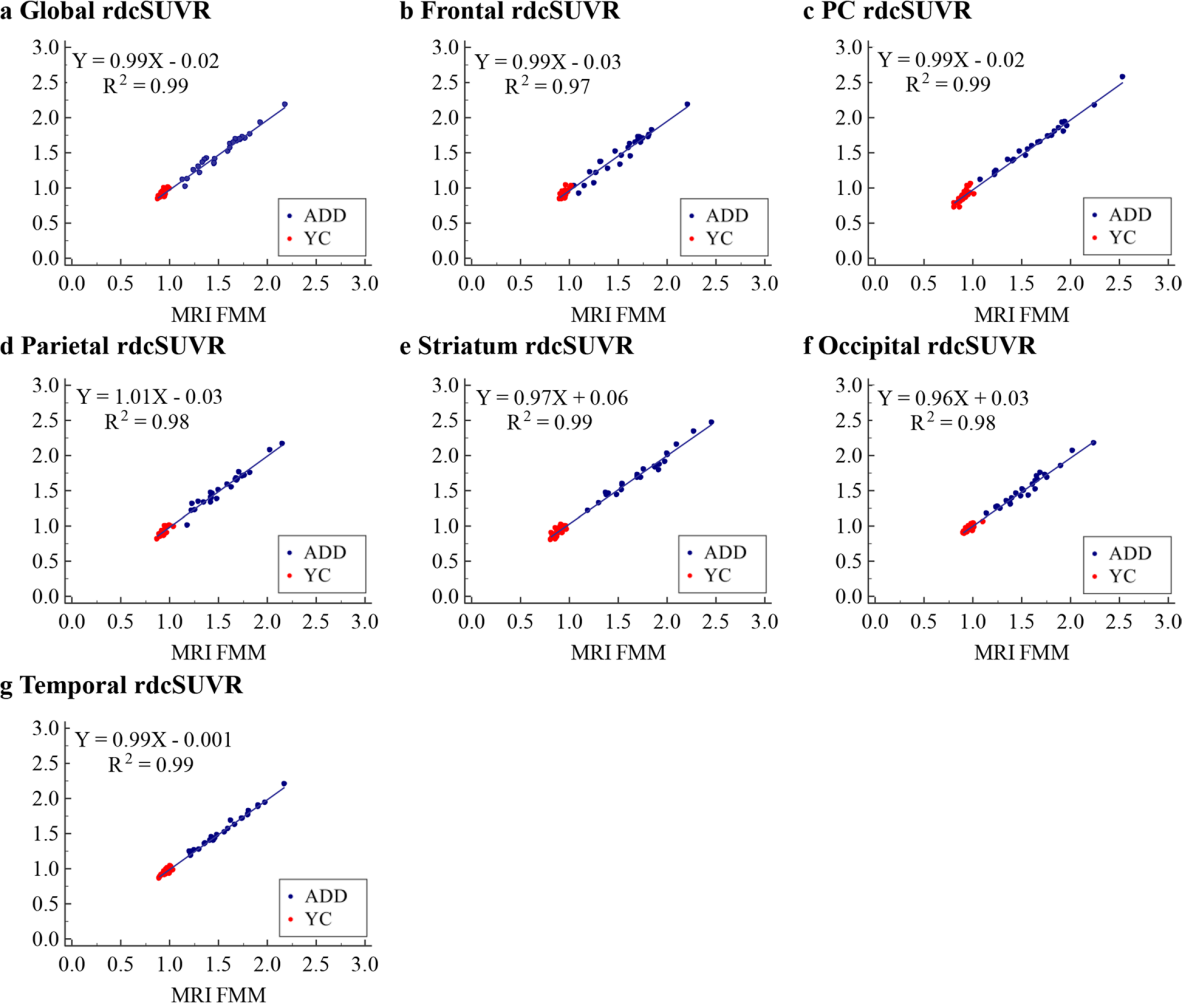
Plots of correlation of global and regional rdcSUVR between MRI-based and CT-based methods for FMM. **a** Global rdcSUVR, **b** Frontal rdcSUVR, **c** PC rdcSUVR, **d** Parietal rdcSUVR, **e** Striatum rdcSUVR, **f** Occipital rdcSUVR, **g** Temporal rdcSUVR. Abbreviations: ADD, Alzheimer’s disease dementia; YC, young control; FMM, ^18^F-flutemetamol; rdcSUVR, standardized uptake value ratio derived from FMM-FBB CTX VOI and regional VOIs; PC, posterior cingulate

**Fig. 3:**
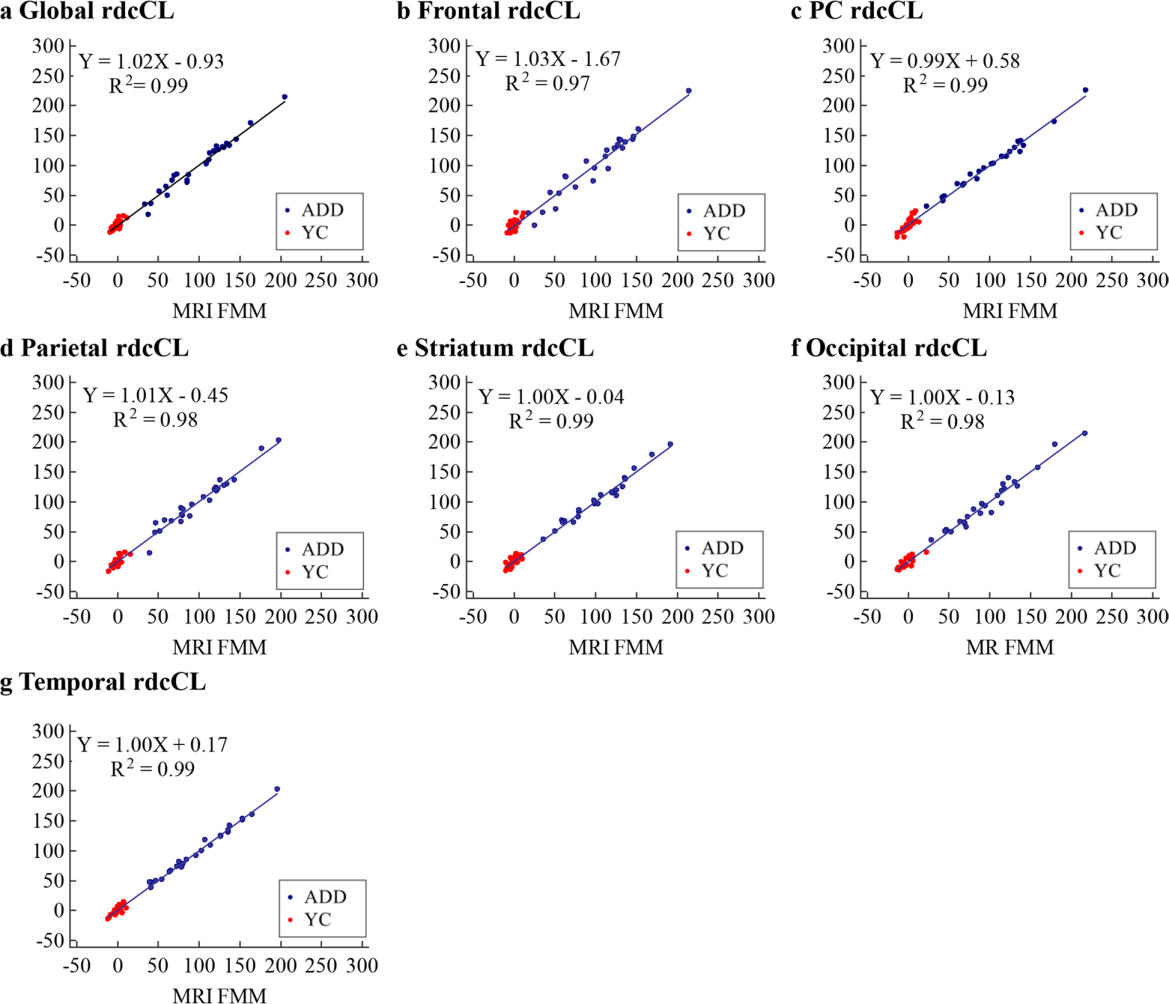
Plots of correlation of FMM rdcCL between MRI-based and CT-based methods. **a** Global rdcCL, **b** Frontal rdcCL, **c** PC rdcCL, **d** Parietal rdcCL, **e** Striatum rdcCL, **f** Occipital rdcCL, **g** Temporal rdcCL. Abbreviations: ADD, Alzheimer’s disease dementia; YC, young control; FMM, ^18^F-flutemetamol; rdcCL, Centiloid scales of FMM-FBB CTX VOI and regional VOIs; PC, posterior cingulate

Both FMM and FBB showed that SUVRs between MRI-based and CT-based methods were highly correlated globally and regionally in the head-to-dead dataset (Fig. 2 and Additional file 1: Fig. S6a; *R*^2^ = 0.97–0.99). For reliability, the correlation between MRI-based and CT-based rdcCLs was investigated. FMM and FBB showed that rdcCLs between MRI-based and CT-based methods were highly correlated (*R*^2^ = 0.97–0.99; Fig. 3 and Additional file 1: Fig. S6b).

**Fig. 4:**
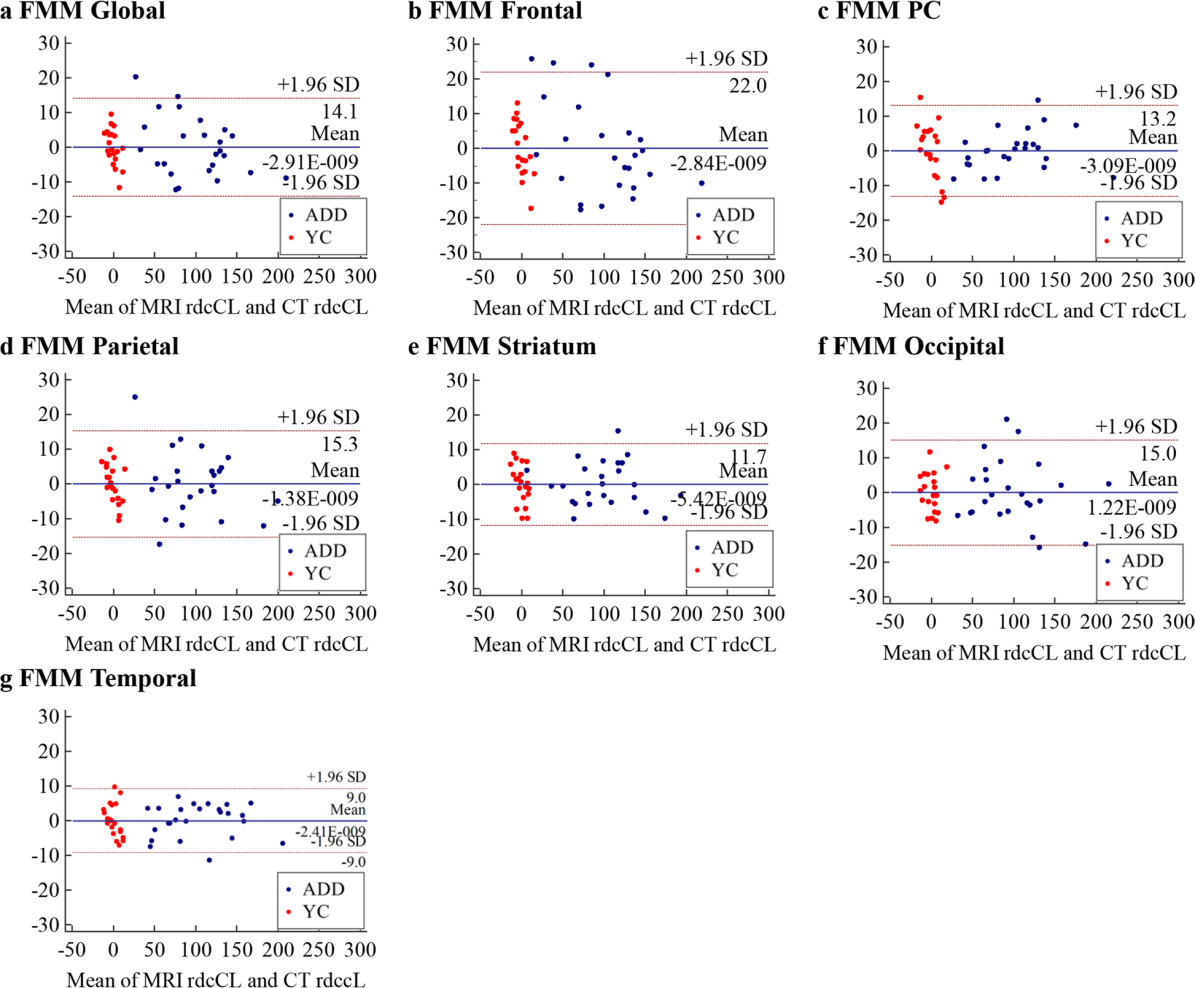
Bland-Altman plots of rdcCLs between MRI-based and CT-based Centiloid methods for FMM. **a** Global rdcCL, **b** Frontal rdcCL, **c** PC rdcCL, **d** Parietal rdcCL, **e** Striatum rdcCL, **f** Occipital rdcCL, **g** Temporal rdcCL. Abbreviations: ADD, Alzheimer’s disease dementia; YC, young control; FMM, ^18^F-flutemetamol; rdcCL, Centiloid scales of FMM-FBB CTX VOI and regional VOIs; PC, posterior cingulate

For precision, the global and regional absolute differences of FMM and FBB rdcCLs between MRI-based and CT-based methods were investigated as shown in Additional file 1: Table S1. Figure 4 shows the distribution plots of absolute FMM rdcCL difference between MRI-based and CT-based methods, which are similar to those of FBB (Additional file 1: Fig. S6c). The GEE results for the absolute difference of rdcCLs between FMM and FBB globally and in six regions showed that the CT-based method was not different from the MRI-based method, globally or regionally (Additional file 1: Table S1 and Additional file 1: Fig. S7).

### Validation of CT-based rdcCLs in independent participants

**Fig. 5:**
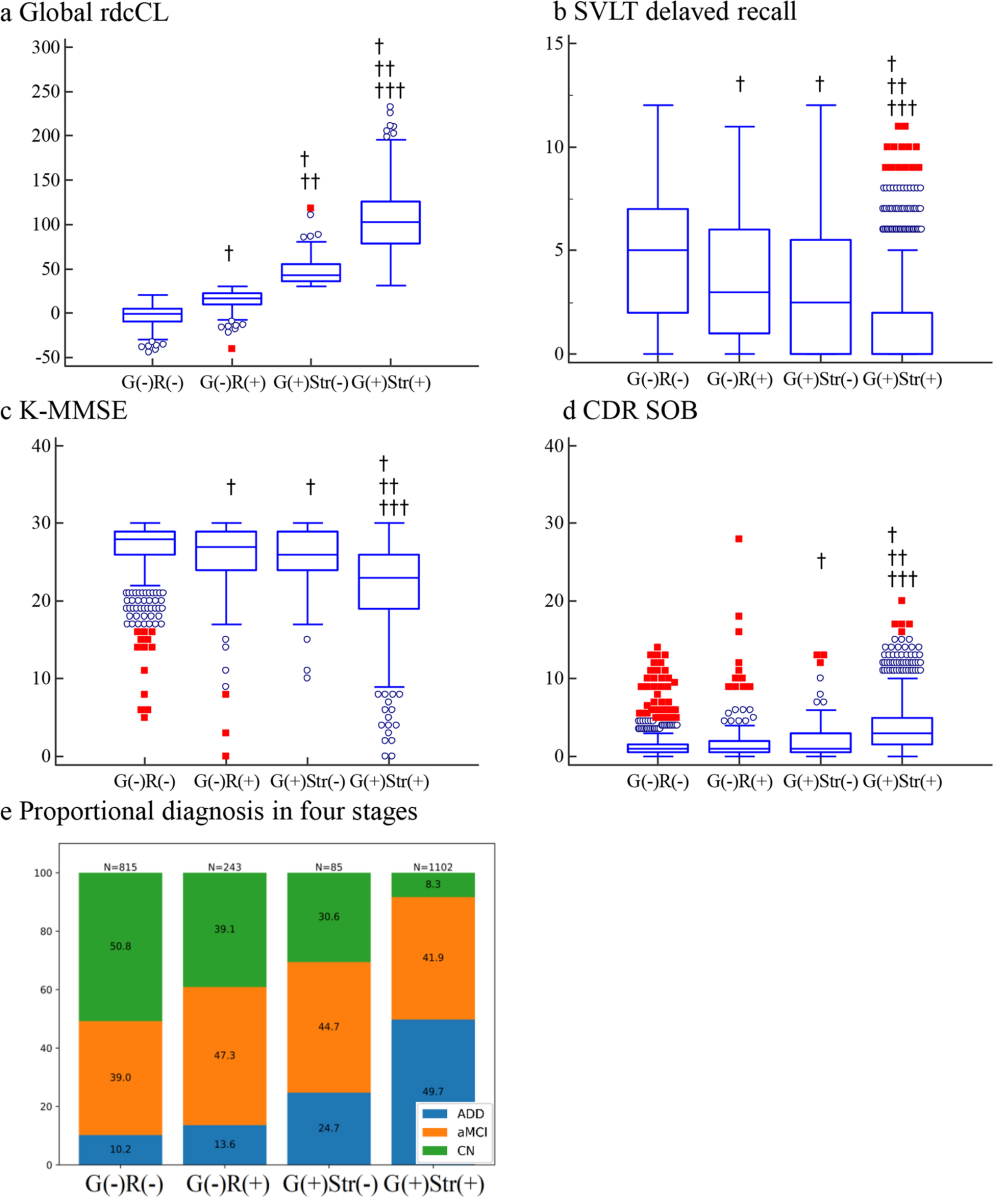
Comparison of the neuropsychological performance classified into four groups based on regional, global, and striatal cutoffs. In **a**–**d**, red squares indicate values higher than the 3rd quartile + 3 × IQR, and blue circles indicate values higher than the 3^rd^ quartile + 1.5 × IQR of a box-and-whisker plot. † *p* < 0.05 group *vs*. G(−)R(−). †† *p* < 0.05 group *vs*. G(−)R(+). ††† *p* < 0.05 group *vs*. G(+)Str(−). Abbreviations: ADD, Alzheimer’s disease dementia; aMCI, amnestic mild cognitive impairment; CN, cognitive normal; rdcCL, Centiloid scales of FMM-FBB CTX VOI and regional VOIs; CDR-SOB, Clinical Dementia Rating Scale Sum of Boxes; MMSE, Mini-Mental State Examination; SVLT, Seoul Verbal Learning Test; G(−)R(−), global (−) and regional (−) Aβ rdcCL scales; G(−)R(+), global (−) and regional (+) Aβ rdcCL scales; G(+)Str(−), global (+) and striatal (−) Aβ rdcCL scales; G(+)Str(+), global (+) and striatal (+) Aβ rdcCL scales; IQR, interquartile range

In our clinical validation study, the average age (SD) of all participants was 69.6 (10.3) years, and 56.1% were females (Table 1). In addition, 32.1% of participants were in the G(−)R(−) group, 11.4% in the G(−)R(+) group, 5.3% in the G(+)Str(−) group, and 52.3% in the G(+)Str(+) group. Figure 5a shows the global rdcCL scales in each group, which tended to increase from the G(−)R(−) to G(+)Str(+) group. The global rdcCL was higher in the G(−)R(+) group than in the G(−)R(−) group (*p* value <0.0001). The SVLT delayed recall score was lower in the G(−)R(+) group than in the G(−)R(−) group (*p* value <0.0001). The MMSE was lower in the G(−)R(+) group than in the G(−)R(−) group (*p* value = 0.0441). The global rdcCL was higher in the G(+)Str(+) group than in the G(+)Str(−) group (*p* value < 0.0001). The SVLT delayed recall and MMSE scores were lower in the G(+)Str(+) group than in the G(+)Str(−) group (*p* value < 0.0001). CDR-SOB was higher in the G(+)Str(+)group than in the G(+)Str(−)group (*p* value < 0.0001) as shown in Fig. 5b–e. The increase in the number of ADD participants based on group order is shown in Fig. 5f.

## Discussion

In the present study, CT-based rdcCL scales of Aβ ligands were developed using head-to-head datasets of FMM and FBB PET ligands, and their clinical efficacy was validated using an independent large-sized cohort. The major findings were as follows: both MRI-based and CT-based rdcCL scales showed that FMM and FBB were highly correlated globally and regionally. In addition, both FMM and FBB showed that CT-based rdcCL scales were mutually highly correlated with MRI-based rdcCL scales, and absolute differences in rdcCL scales between CT-based and MRI-based methods were not significant; the G(−)R(+) and G(+)Str(+) groups predicted worse neuropsychological performance than the G(−)R(−) and the G(+)Str(−) groups. Collectively, the results indicate that it is feasible to convert FMM or FBB rdcSUVR values into rdcCL scales regionally without additional MRI scans. Such a method could be more accessible to researchers compared to other approaches and would be applicable to a variety of different conditions.

The first major finding of this study was that both MRI-based and CT-based rdcCL scales showed FMM and FBB to be mutually highly correlated, globally and regionally. This indicates that the CT-based method can harmonize FMM and FBB ligands without paired MRI data. In a previous study by Lilja et al., a PET-only normalization method was developed to resolve variability of Aβ uptake in FMM PET [20]. The present study was proposed because MRI images might not always be available in the analysis of Aβ PET uptake. Furthermore, in a recent study from the AIBL group, global CL scales were developed using PET templates without MRI images [21]. Since our common target regions included parts of the occipital region, we calculated rdcCL in the occipital region. This has some clinical implication because cerebrovascular disease might be related to increased Aβ uptake in the occipital region [22, 23]. However, the method developed in the present study provides regional as well as global rdcCL scales in the six regions including the striatum. To the best of our knowledge, this is the first study in which CT-based rdcCL scales have been developed.

Second, in terms of reliability, both FMM and FBB showed CT-based rdcCL scales to be highly correlated with MRI-based rdcCL scales. In particular, all regional VOIs showed CT-based rdcCL scales to be highly correlated with MRI-based rdcCL scales. In terms of precision, absolute differences in rdcCL scales between CT-based and MRI-based methods were not significant; most of the absolute differences applied to the head-to-head cohort in all ligands were located within the significant lines of the Bland-Altman graphs. Furthermore, because absolute differences in CT-based rdcCL scales between FMM and FBB were not larger than in MRI-based methods, our CT-based rdcCL scale is a reasonable method to convert regional FMM or FBB rdcSUVRs into rdcCL scales, at least in environments where MRI data are not available and regional Aβ uptake information is needed.

The final major finding in the clinical validation of the CT-based rdcCL scales was that the G(−)R(+) group had worse neuropsychological performance than the G(−)R(−) group. This finding was consistent with a previous study in AD-related cognitive impairment (ADCI), showing that subjects with regionally increased Aβ uptake had worse memory and hippocampal changes than subjects without regionally increased Aβ uptake [8]. Furthermore, in the present study, the G(+)Str(+) group had worse neuropsychological performance than the G(+)Str(−) group, which is in agreement with a previous study in 2018 from our group showing striatal involvement of Aβ as a predictor of poor prognosis. Previously, researchers were unable to detect subjects with regionally increased Aβ uptake except in individuals with subthreshold global amyloid level or striatal involvement of Aβ because the conventional dcCL scale only provided information regarding global Aβ level, not regional Aβ level. Therefore, CT-based rdcCL scales used in the present study might provide clinicians with more sensitive diagnostic and prognostic data.

One strength of the study was that a prospectively well-designed head-to-head dataset of FMM and FBB PET ligands was used to develop CT-based rdcCL scales of Aβ ligands. Another strength was that clinical efficacy of the CT-based rdcCL scales was validated in an independent, large-sized, carefully phenotyped cohort using non-invasive amyloid imaging and neuropsychological performance.

### Limitations

The present study had several limitations. First, the standard for the presence or absence of Aβ uptake in each brain region has not been established. Thus, further regional pathologic verifications are needed for more realistic cutoffs. Second, because FBB is derived from Congo red [24] and FMM is based on the chemical structure of thioflavin T [25], these two ligands possibly show different dynamic ranges or uptake in the cortex, striatum, and white matter. Although the two ligands are comparable for imaging AD pathology in vivo, FMM might be better than FBB for detecting amyloid burden in the striatum. Third, a partial volume correction (PVC) was not applied to our dataset. Fourth, since we did not convert values between the FMM and FBB ligands, there might be some debate as to whether our rdcCL scales could harmonize the two ligands. However, this argument might be mitigated by our findings of excellent correlations between the FMM and FBB rdcCL scales (*R*^2^ > 0.96, slope 0.99–1.03; Additional file 1: Fig. S5). Considering that the slope between FMM and FBB Klunk’s CL values was 0.79, our rdcCL methods are acceptable to harmonize quantitative FMM and FBB uptakes [6]. However, since differences between the FMM and FBB rdcCL scales were noted in a given subject with higher amyloid uptakes (Additional file 1: Fig. S7), we should be cautious in interpreting our results in a given subject with higher amyloid uptakes. Also, since our cutoff values of rdcCL were obtained from the dcCL method, those could not be compared to standard CL scales of the standard Klunk CL method. Fifth, there were differences in the regional cut-off values between FBB and FMM ligands (Additional file 1: Table S2). However, the AUCs of the results applying these cut-off values to the visual assessment results were very high, mostly 0.9 or high. Finally, our CTs were low-dose and only were acquired for attenuation correction. The actual anatomical information in those is limited. However, in the present study, our CT-based rdcCLs were comparable to MRI-based rdcCLs in terms of reliability and precision. Our method provided information of rdcCL scales of Aβ ligands without MRI data, which can provide clinicians with a better understanding of biomarker-guided diagnosis and prediction of prognosis.

## Conclusions

In conclusion, our CT-based rdcCL scales method was comparable to the MRI-based method. Furthermore, compared to the conventional CL method, the information of our method was able to better predict poor clinical impairment.

## Supplementary Information

**Additional file 1: Supplementary Methods 1.** Three-dimensional (3D) T1 parameters. **Supplementary Methods 2.** The Seoul Neuropsychological Screening Battery 2nd edition (SNSB-II). **Supplementary Methods 3.** MRI-based rdcCLs. **Table S1.** GEE using the absolute difference FMM and FBB rdcCL between CT-based and MRI-based methods. **Table S2.** Regional cut-off values of FBB and FMM ligands. **Figure S1.** Regional visual positivity against regional MRI- and CT-based rdcCL scales for **a** FMM and **b** FBB PET. **Figure S2.** MRI-based Aβ uptake patterns in ADD cohort compared with OCs in a FMM and b FBB. a and b show regions in which the ADD cohort had higher rdcSUVR differences than OCs for FMM and FBB, respectively. c MRI-based FMM-FBB regional VOIs and f CT-based FMM-FBB regional VOIs were mapped onto the MNI-152 template. CT-based Aβ uptake patterns in the ADD cohort compared with those of OCs in d FMM and e FBB. d and e show regions in which the ADD cohort had higher SUVR differences than those of OCs for FMM and FBB, respectively. g The intersectional region between MR- and CT-based methods (red), voxels included in the only-MRI-based method (light blue), and voxels included in the only-CT-based method (light yellow). **Figure S3.** Plots of correlation of rdcSUVR between a MRI-based and b CT-based methods for FMM and FBB PET in the head-to-head cohort, globally and regionally. **Figure S4.** Plots of MRI-based conversion of rdcSUVR into rdcCL globally and in six regions a FMM and b FBB and plots of CT-based conversion in c FMM and d FBB and global and regional equations of linear regression. **Figure S5.** Correlation plots of rdcCL between PET ligands in a MRI-based and b CT-based rdcCL in the head-to-head cohort, globally and regionally. **Figure S6.** Correlation plots of a rdcSUVR and b rdcCL between rdcCL methods and Bland-Altman plots of rdcCL between **c** the methods for FBB PET. **Figure S7.** Plots of difference in rdcCLs between PET ligands for a MRI-based and b CT-based methods in the head-to-head cohort.

## Data Availability

Requests for resources, reagents, and further information will be made available from the lead corresponding author (Sang Won Seo) on reasonable request.

## Abbreviations

Aβ: β-amyloid
CT: Computed tomography
AUC: Area under the curve
PET: Positron emission tomography
MRI: Magnetic resonance imaging
11C-PiB: 11C-labeled Pittsburgh compound B
rdcSUVR: Standardized uptake value ratio derived from FMM-FBB CTX VOI and regional VOIs
rdcCL: Centiloid scales of FMM-FBB CTX VOI and regional VOIs
FMM: 18F-flutemetamol
FBB: 18F-florbetaben
ADD: Alzheimer’s disease dementia
YC: Young controls
OC: Old controls
NIA-AA: National Institute on Aging-Alzheimer’s Association
APOE-ε4: Apolipoprotein E ε4 allele
MMSE: Mini-Mental State Examination
G(−)R(−): Global (−) and regional (−) Aβ rdcCL scales
G(−)R(+): Global (−) and regional (+) Aβ rdcCL scales
G(+)Str(−): Global (+) and striatal (−) Aβ rdcCL scales
G(+)Str(+): Global (+) and striatal (+) Aβ rdcCL scales
PC: Posterior cingulate
aMCI: Amnestic mild cognitive impairment
CN: Cognitive normal
CDR-SOB: Clinical Dementia Rating Scale Sum of Boxes
SVLT: Seoul Verbal Learning Test
