# Supplementary material for: Development and clinical validation of CT-based regional modified Centiloid method for amyloid PET
Source: Alzheimers Res Ther. 2022 Oct 20;14:157. doi: 10.1186/s13195-022-01099-0 (PMC9585745; doi:10.1186/s13195-022-01099-0)
Supplement: Supplementary file 1 — Additional file 1: Supplementary Methods 1. Three-dimensional (3D) T1 parameters. Supplementary Methods 2. The Seoul Neuropsychological Screening Battery 2nd edition (SNSB-II). Supplementary Methods 3. MRI-based rdcCLs. Table S1. GEE using the absolute difference FMM and FBB rdcCL between CT-based and MRI-based methods. Table S2. Regional cut-off values of FBB and FMM ligands. Figure S1. Regional visual positivity against regional MRI- and CT-based rdcCL scales for a FMM and b FBB PET. Figure S2. MRI-based Aβ uptake patterns in ADD cohort compared with OCs in a FMM and b FBB. a and b show regions in which the ADD cohort had higher rdcSUVR differences than OCs for FMM and FBB, respectively. c MRI-based FMM-FBB regional VOIs and f CT-based FMM-FBB regional VOIs were mapped onto the MNI-152 template. CT-based Aβ uptake patterns in the ADD cohort compared with those of OCs in d FMM and e FBB. d and e show regions in which the ADD cohort had higher SUVR differences than those of OCs for FMM and FBB, respectively. g The intersectional region between MR- and CT-based methods (red), voxels included in the only-MRI-based method (light blue), and voxels included in the only-CT-based method (light yellow). Figure S3. Plots of correlation of rdcSUVR between a MRI-based and b CT-based methods for FMM and FBB PET in the head-to-head cohort, globally and regionally. Figure S4. Plots of MRI-based conversion of rdcSUVR into rdcCL globally and in six regions a FMM and b FBB and plots of CT-based conversion in c FMM and d FBB and global and regional equations of linear regression. Figure S5. Correlation plots of rdcCL between PET ligands in a MRI-based and b CT-based rdcCL in the head-to-head cohort, globally and regionally. Figure S6. Correlation plots of a rdcSUVR and b rdcCL between rdcCL methods and Bland-Altman plots of rdcCL between c the methods for FBB PET. Figure S7. Plots of difference in rdcCLs between PET ligands for a MRI-based and b CT-based methods in the head-to-head c [file 13195_2022_1099_MOESM1_ESM.docx]

**Development and Clinical Validation of CT-based Regional modified Centiloid Method for Amyloid PET**

**Supplementary Methods 1.** Three-dimensional (3D) T1 parameters

Three-dimensional (3D) T1 parameters were as follows: sagittal slice thickness, 1.0 mm over contiguous slices with 50% overlap; no gap; repetition time of 9.9 msec; echo time of 4.6 msec; flip angle of 8°; matrix size of 240 × 240 pixels reconstructed to 480 × 480 over a field of view of 240 mm.

**Supplementary Methods 2.** The Seoul Neuropsychological Screening Battery 2nd edition (SNSB-II)

The Seoul Neuropsychological Screening Battery 2nd edition (SNSB-II) consists of the following five cognitive domains: 1) Memory: the Seoul Verbal Learning Test (SVLT) delayed recall (verbal memory) and Rey-Osterrieth Complex Figure Test (RCFT) delayed recall (visual memory); 2) Language: Korean version of the Boston Naming Test (K-BNT); 3) Visuospatial function: RCFT Copying Test; 4) Frontal executive function: animal and phonemic portion of the Controlled Oral Word Association Test (COWAT) and the Stroop Test (color reading); 5) Attention: Digit Span Test backward.

**Supplementary Methods 3.** MRI-based rdcCLs

The group-averaged differences between the alzheimer’s disease dementia (ADD) participants and old controls (OCs) using ^18^F-flutemetamol (FMM) (Figure S1a) were similar to the differences using ^18^F-florbetaben (FBB) (Figure S1b). The regions of FMM-FBB CTX VOIs were located mostly in the lateral frontal, lateral temporal, lateral and medial parietal, insular, posterior cingulate, lateral occipital, and anterior ventral striatal regions (Figure S1c). The FMM and FBB rdcSUVR values of the head-to-head cohort showed excellent linear correlation (R^2^ = 0.96–0.97) in the global region and six regional VOIs (Figure S2a).

The regression equations were calculated to convert the FMM rdcSUVR into FMM rdcCL (Figure S3a) and the FBB rdcSUVR into FBB rdcCL (Figure S3b), globally and regionally. Based on direct comparison of the MRI-based method, the rdcCL scales between FMM and FBB of head-to-head PET scans were highly correlated, both globally and regionally (R^2^ = 0.96–0.97; Figure S4a).

**Supplementary Tables**

**Table S1.** GEE using the absolute difference FMM and FBB rdcCL between CT-based and MRI-based methods

| **Mean (SE)** | **CT-based method** | **MRI-based method** | ***p* value** |
| --- | --- | --- | --- |
| Global | 6.16 (1.02) | 6.26 (0.99) | 0.82 |
| Frontal | 7.34 (1.19) | 6.69 (1.14) | 0.21 |
| PC | 7.19 (0.79) | 6.16 (0.82) | 0.07 |
| Parietal | 6.93 (0.92) | 6.41 (0.95) | 0.37 |
| Striatum | 7.12 (1.12) | 7.04 (1.16) | 0.9 |
| Occipital | 7.15 (0.85) | 7.21 (0.97) | 0.95 |
| Temporal | 6.33 (1.01) | 6.45 (1.02) | 0.66 |

Abbreviations: GEE, generalized estimating equation; SE, standard error; FBB, ^18^F-florbetaben; FMM, ^18^F-flutemetamol; rdcCL, Centiloid scales of FMM-FBB CTX VOI and regional VOIs; PC, posterior cingulate

**Table S2.** Regional cut-off values of FBB and FMM ligands.

| **FBB rdcCL** | Global | PC | Frontal | Parietal | Temporal | Striatum |
| --- | --- | --- | --- | --- | --- | --- |
| AUC | 0.96 | 0.92 | 0.87 | 0.95 | 0.96 | 0.89 |
| Optimal cutoff | 30.52 | 50.64 | 17.14 | 33.21 | 25.54 | 40.1 |
| Youden index J | 0.8 | 0.78 | 0.66 | 0.84 | 0.87 | 0.69 |
| **FMM rdcCL** | Global | PC | Frontal | Parietal | Temporal | Striatum |
| AUC | 0.92 | 0.97 | 0.92 | 0.92 | 0.92 | 0.93 |
| Optimal cutoff | 38.67 | 40.71 | 13.99 | 58.75 | 43.3 | 61.56 |
| Youden index J | 0.78 | 0.85 | 0.77 | 0.73 | 0.73 | 0.75 |

Abbreviations: AUC, Area under the curve; FMM, ^18^F-flutemetamol; FBB, ^18^F-florbetaben; rdcCL, Centiloid scales of FMM-FBB CTX VOI and regional VOIs; PC, posterior cingulate

**Supplementary Figures**

**
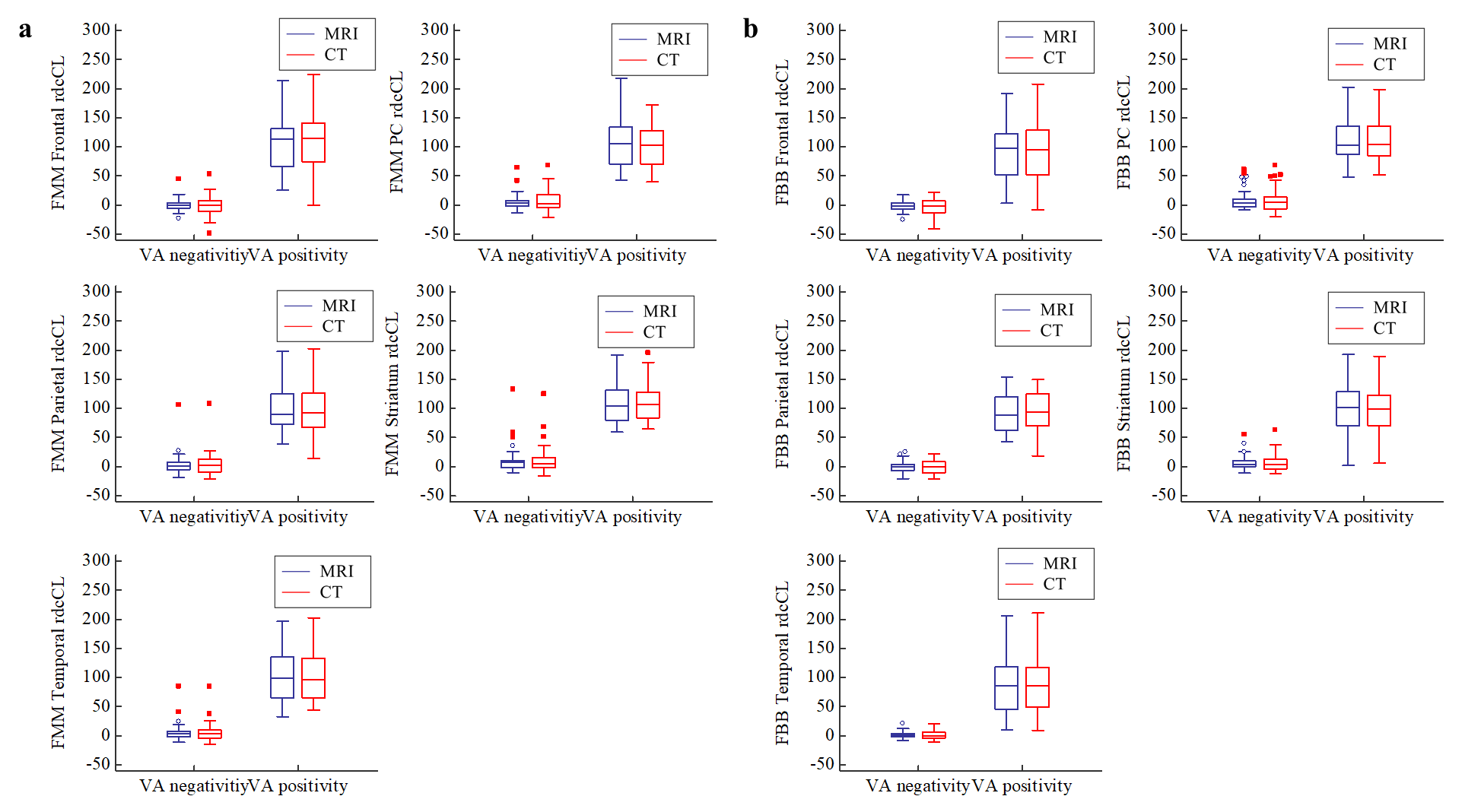
**

**Figure S1.** Regional visual positivity against regional MRI- and CT-based rdcCL scales for **a** FMM and **b** FBB PET.

Abbreviations: FMM, ^18^F-flutemetamol; FBB, ^18^F-florbetaben; rdcCL, Centiloid scales of FMM-FBB CTX VOI and regional VOIs; PC, posterior cingulate


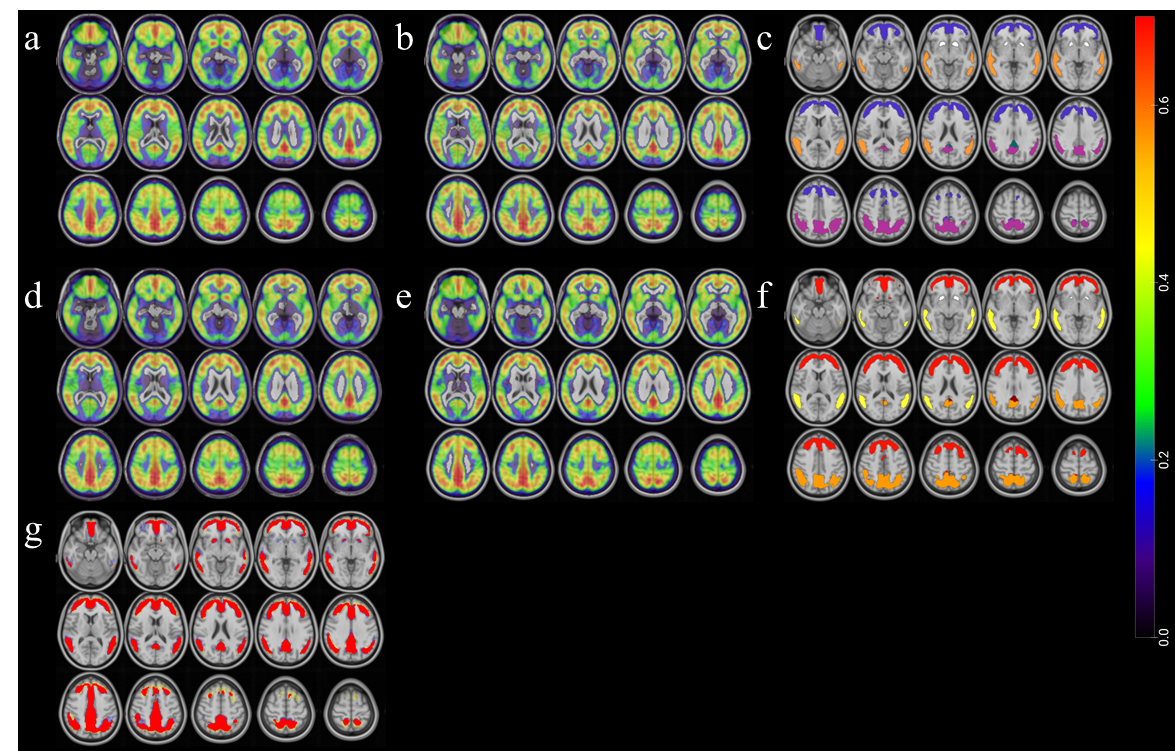


**Figure S2.** MRI-based Aβ uptake patterns in the ADD cohort compared with OCs in **a** FMM and **b** FBB. **a** and **b** show regions in which the ADD cohort had higher rdcSUVR differences than OCs for FMM and FBB, respectively. **c** MRI-based FMM-FBB regional VOIs and **f** CT-based FMM-FBB regional VOIs were mapped onto an MNI-152 template. CT-based Aβ uptake patterns in the ADD cohort compared with OCs in **d** FMM and **e** FBB. **d** and **e** show regions in which the ADD cohort had higher SUVR differences than OCs for FMM and FBB, respectively. **g** Intersectional region between MR- and CT-based methods (red), voxels included in only the MRI-based method (light blue), voxels included in only the CT-based method (light yellow).


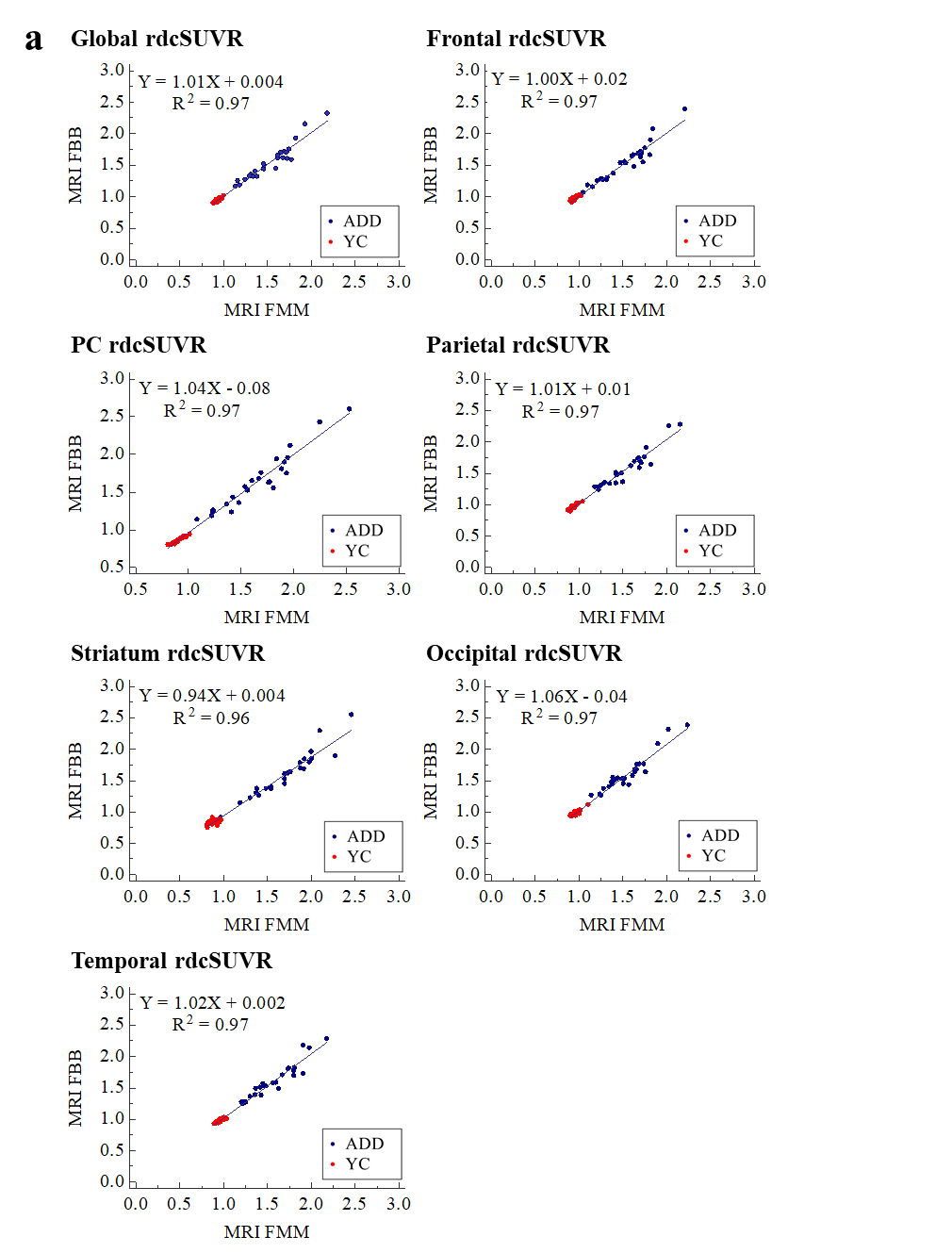

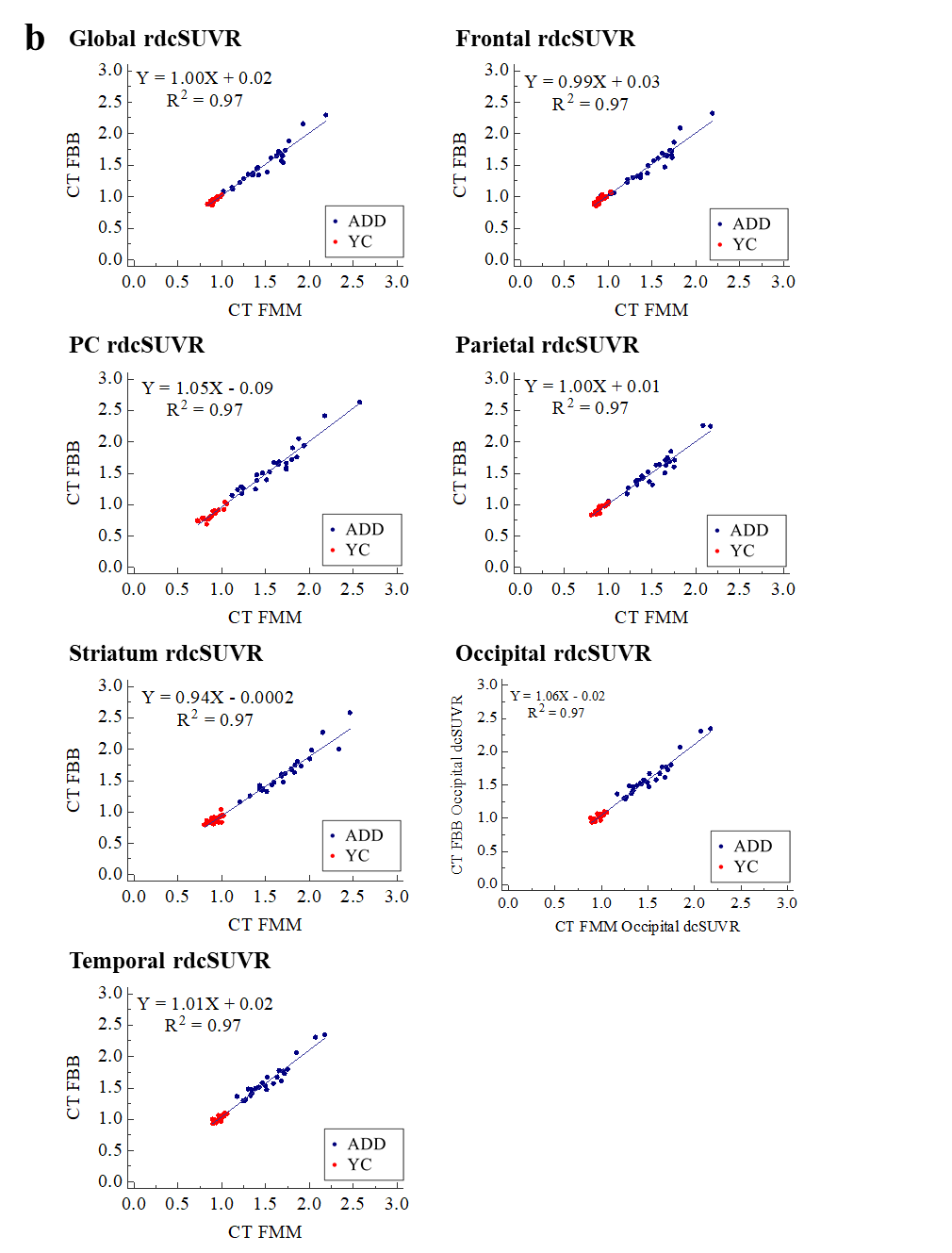


**Figure S3.** Plots of correlation of rdcSUVR with FMM and FBB PET between **a** MRI-based and **b** CT-based methods in the head-to-head cohort, globally and regionally.

Abbreviations: ADD, Alzheimer’s disease dementia; YC, younger control; FBB, ^18^F-florbetaben; FMM, ^18^F-flutemetamol; rdcSUVR, standardized uptake value ratio derived from FMM-FBB CTX VOI and regional VOIs; PC, posterior cingulate


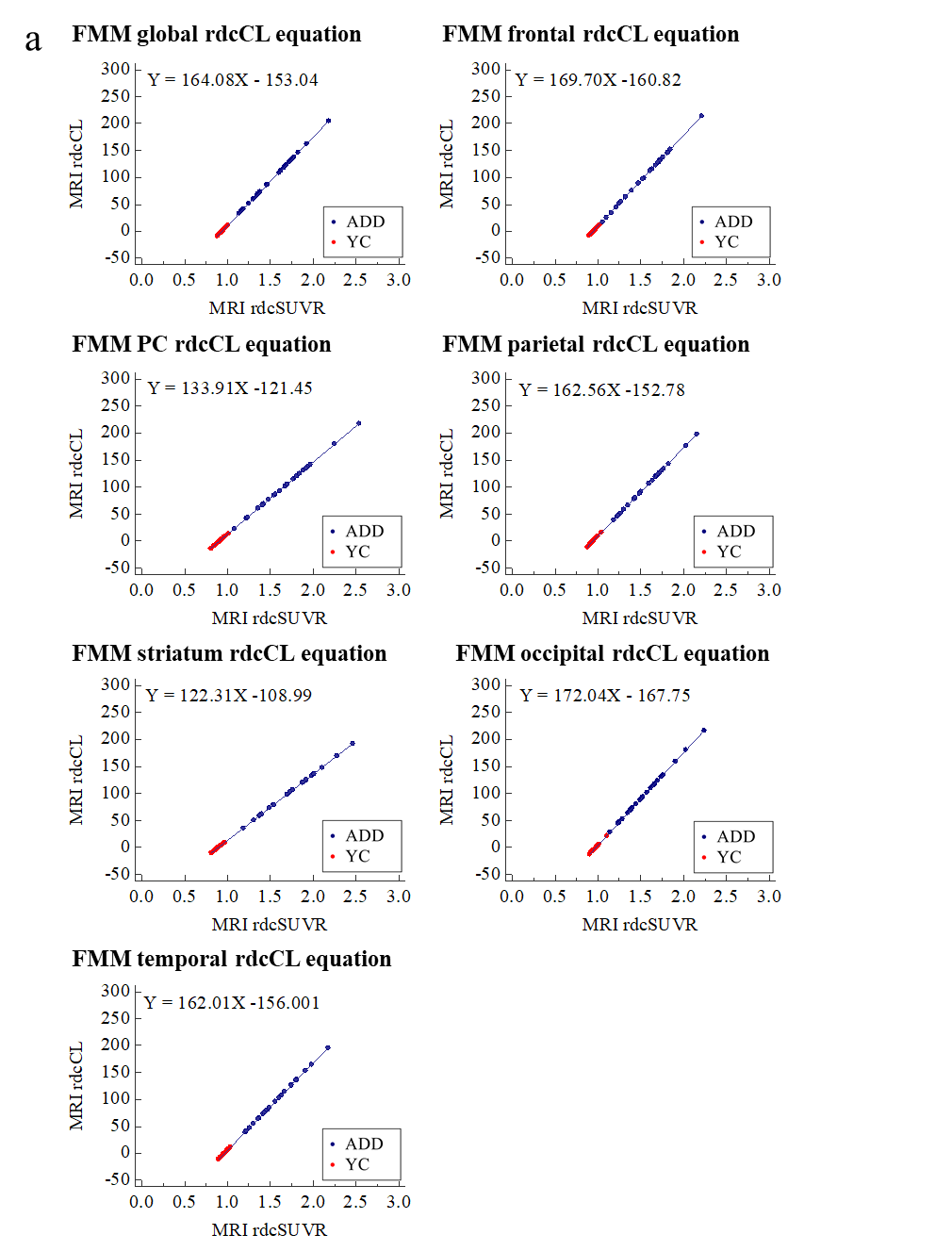

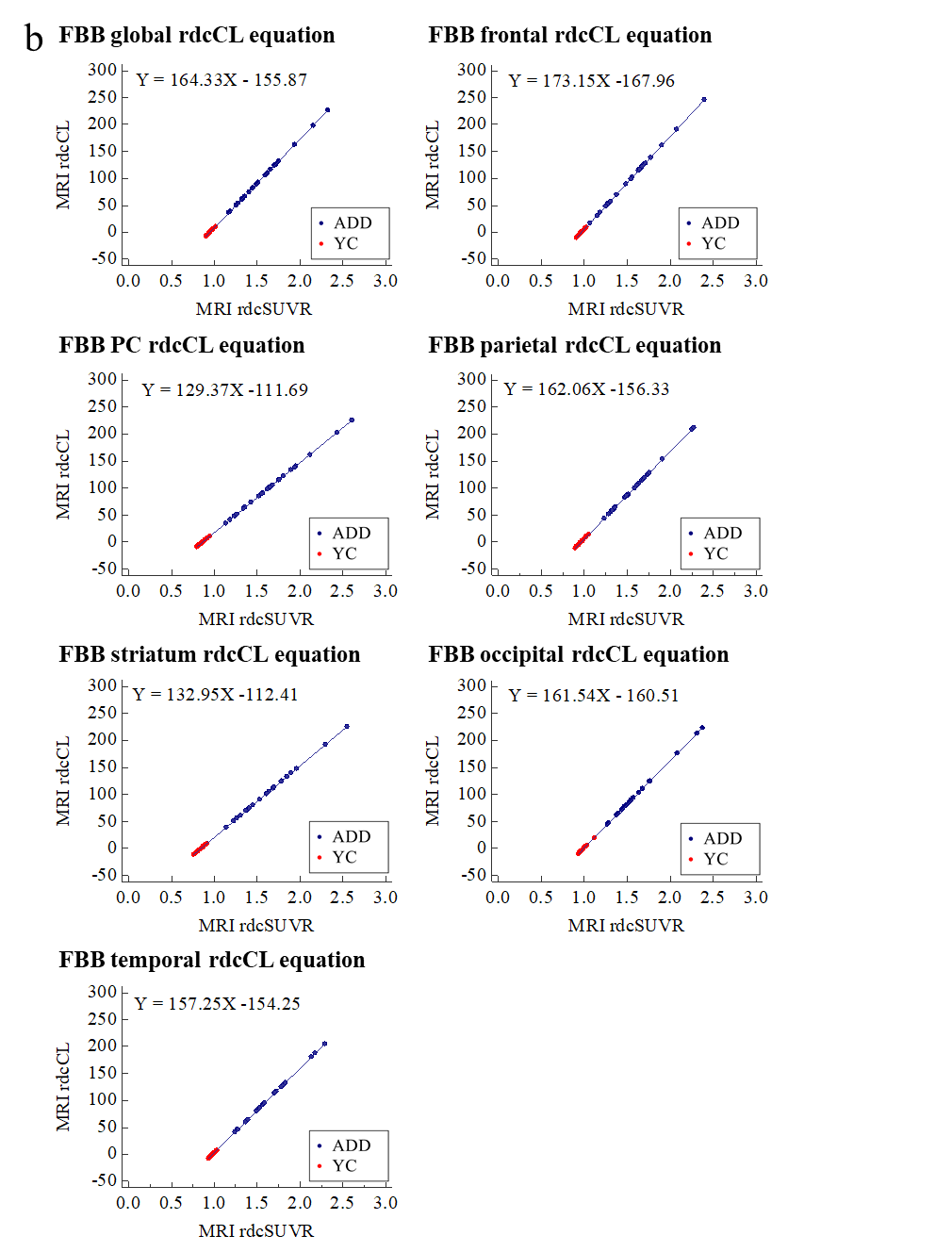

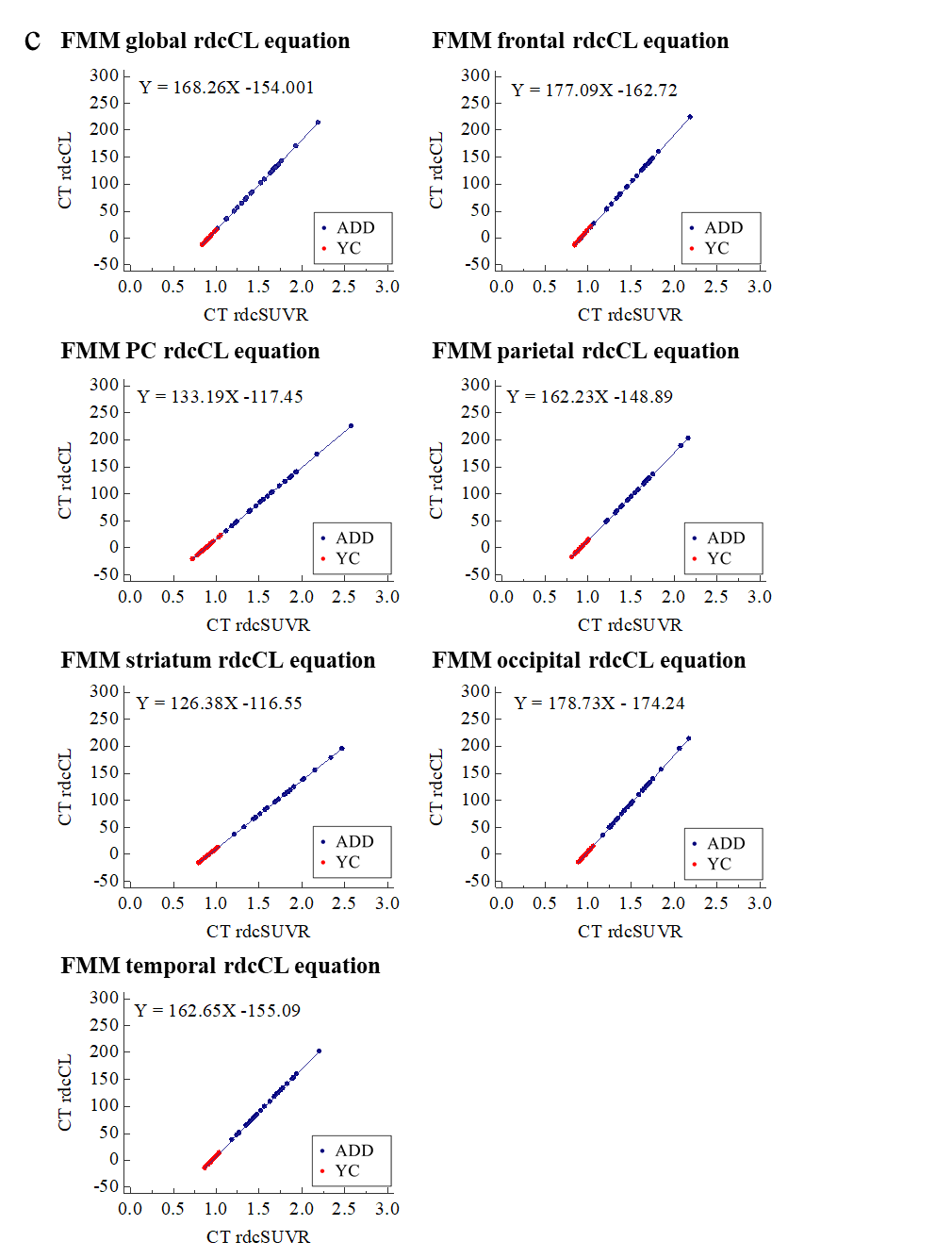

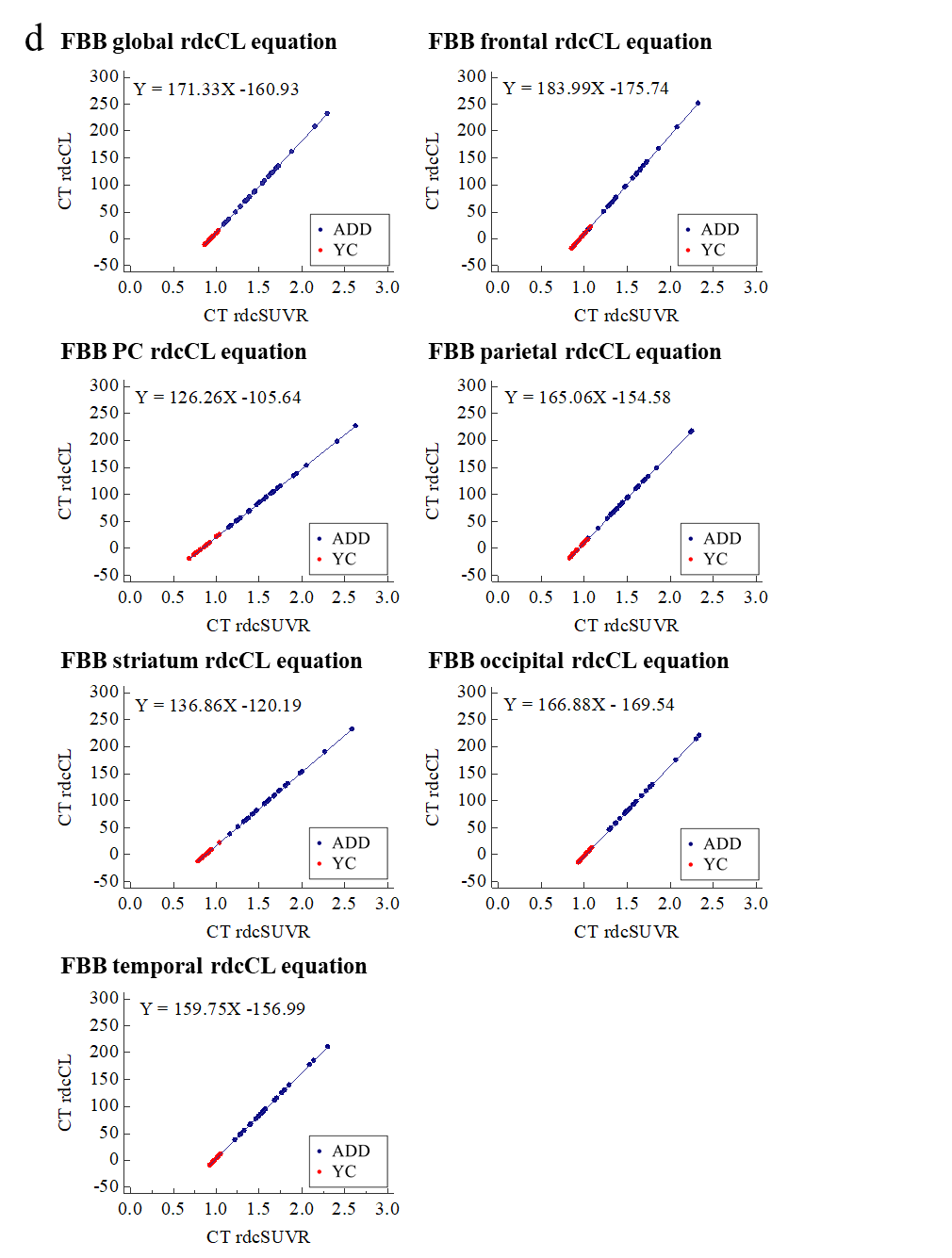


**Figure S4.** Plots of MRI-based conversion of rdcSUVR into rdcCL globally and in six regions **a** FMM and **b** FBB and plots of CT-based **c** FMM and **d** FBB and global and regional equations of linear regression.

Abbreviations: ADD, Alzheimer’s disease dementia; YC, younger control; FMM, ^18^F-flutemetamol; FBB, ^18^F-florbetaben; rdcSUVR, standardized uptake value ratio derived from FMM-FBB CTX VOI and regional VOIs; rdcCL, Centiloid scales of FMM-FBB CTX VOI and regional VOIs; PC, posterior cingulate


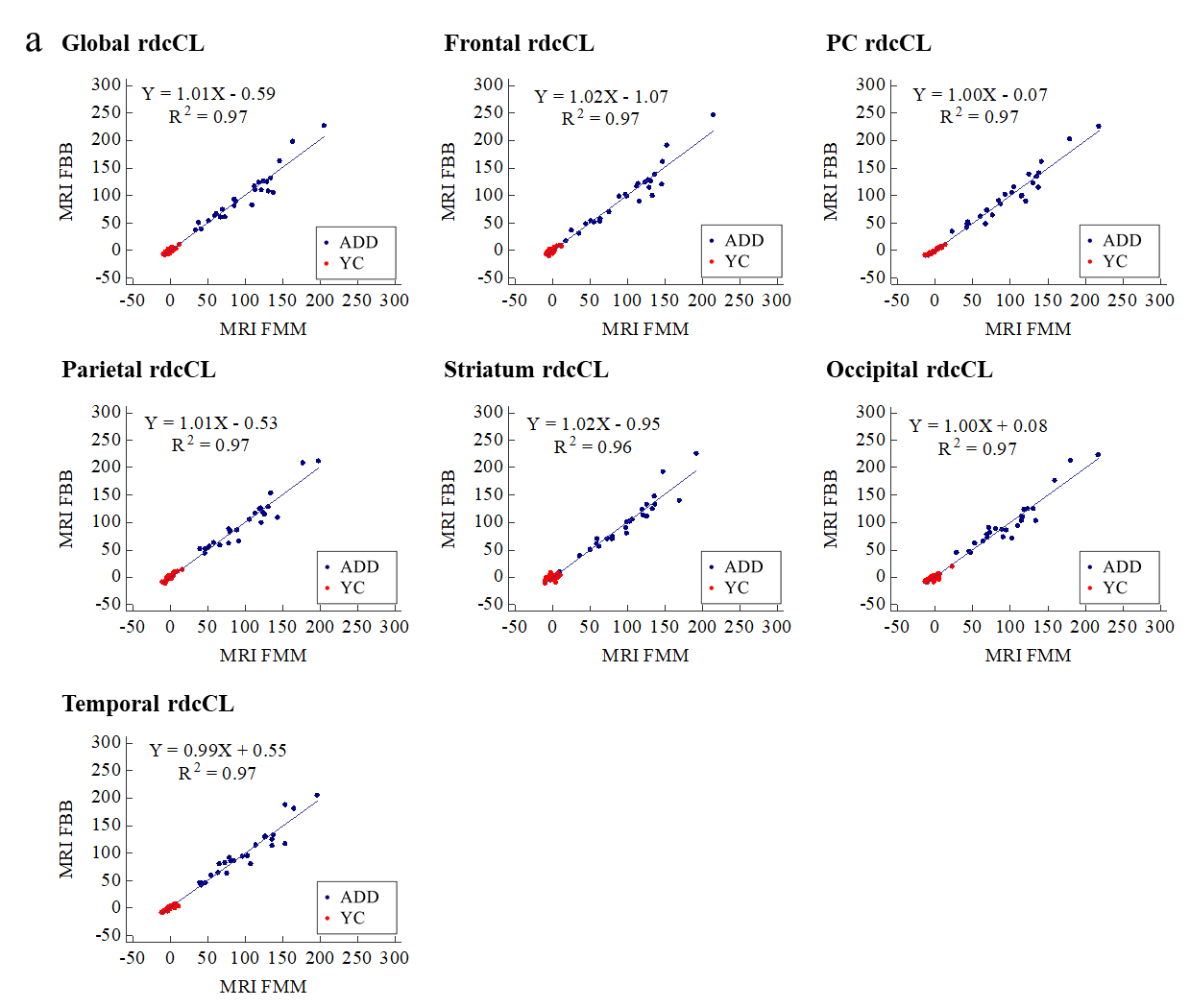

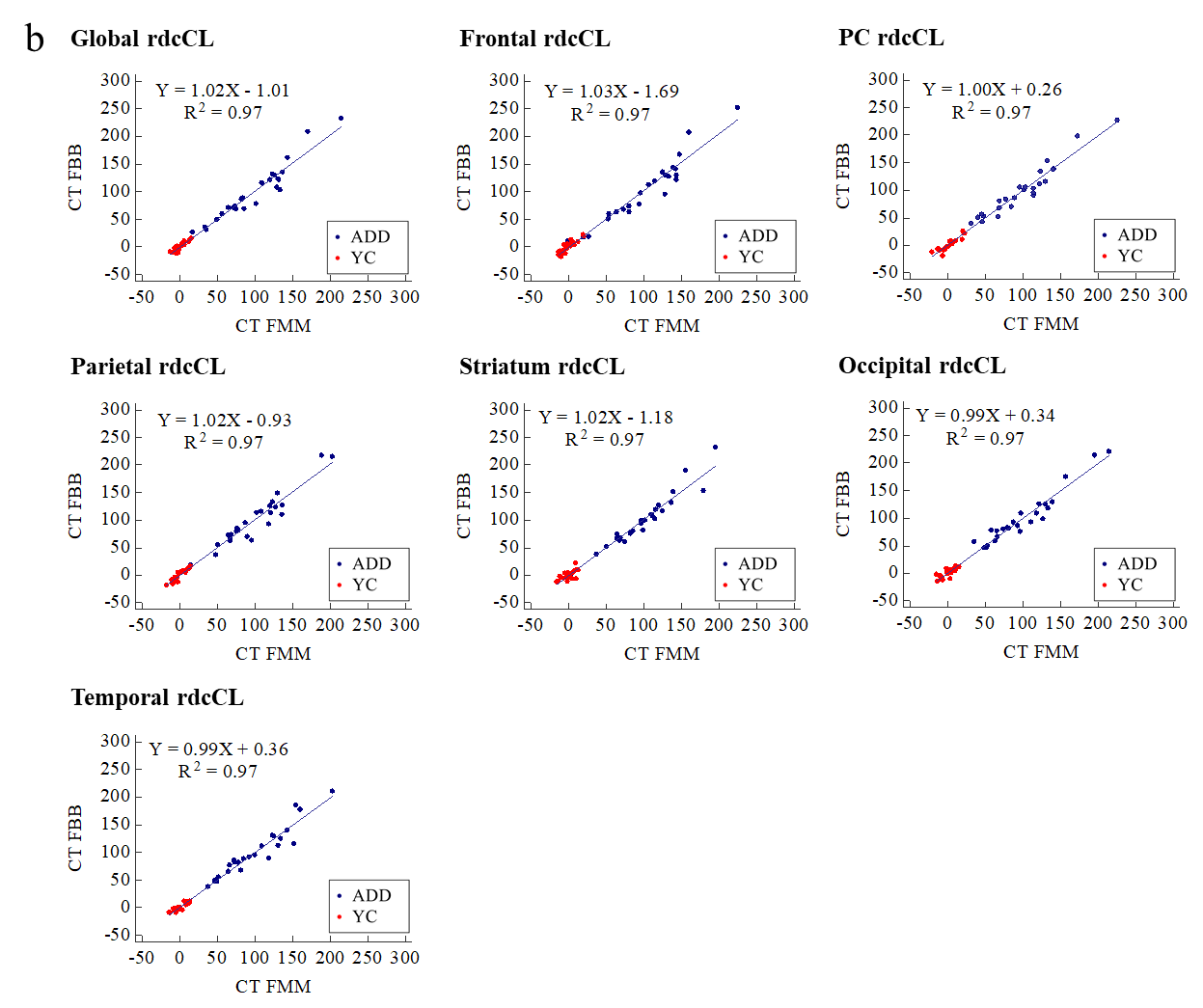


**Figure S5.** Correlation plots of rdcCL between PET ligands in **a** MRI-based and **b** CT-based rdcCL in the head-to-head cohort, globally and regionally.

Abbreviations: ADD, Alzheimer’s disease dementia; YC, younger control; FMM, ^18^F-flutemetamol; FBB, ^18^F-florbetaben; rdcCL, Centiloid scales of FMM-FBB CTX VOI and regional VOIs; PC, posterior cingulate


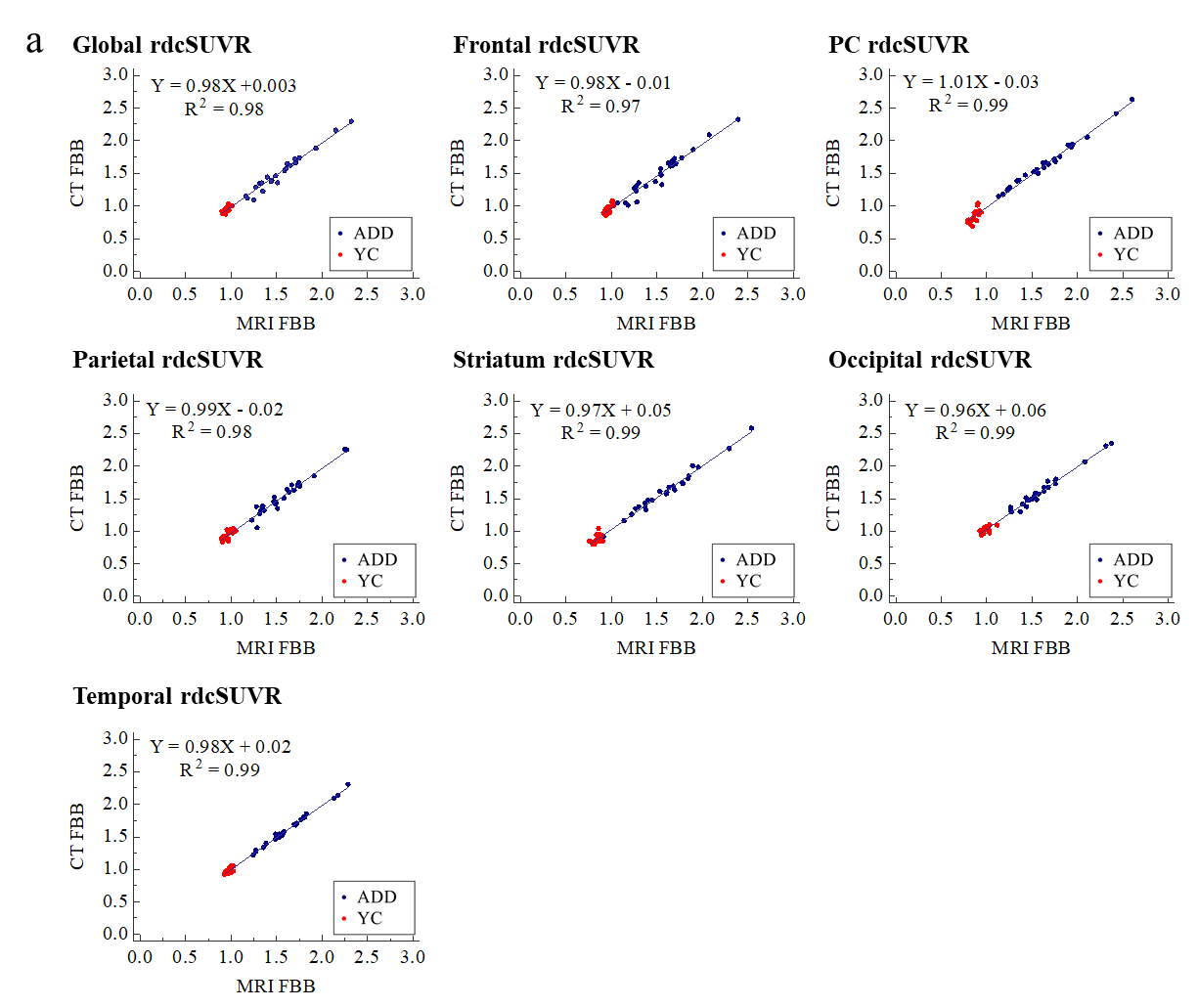

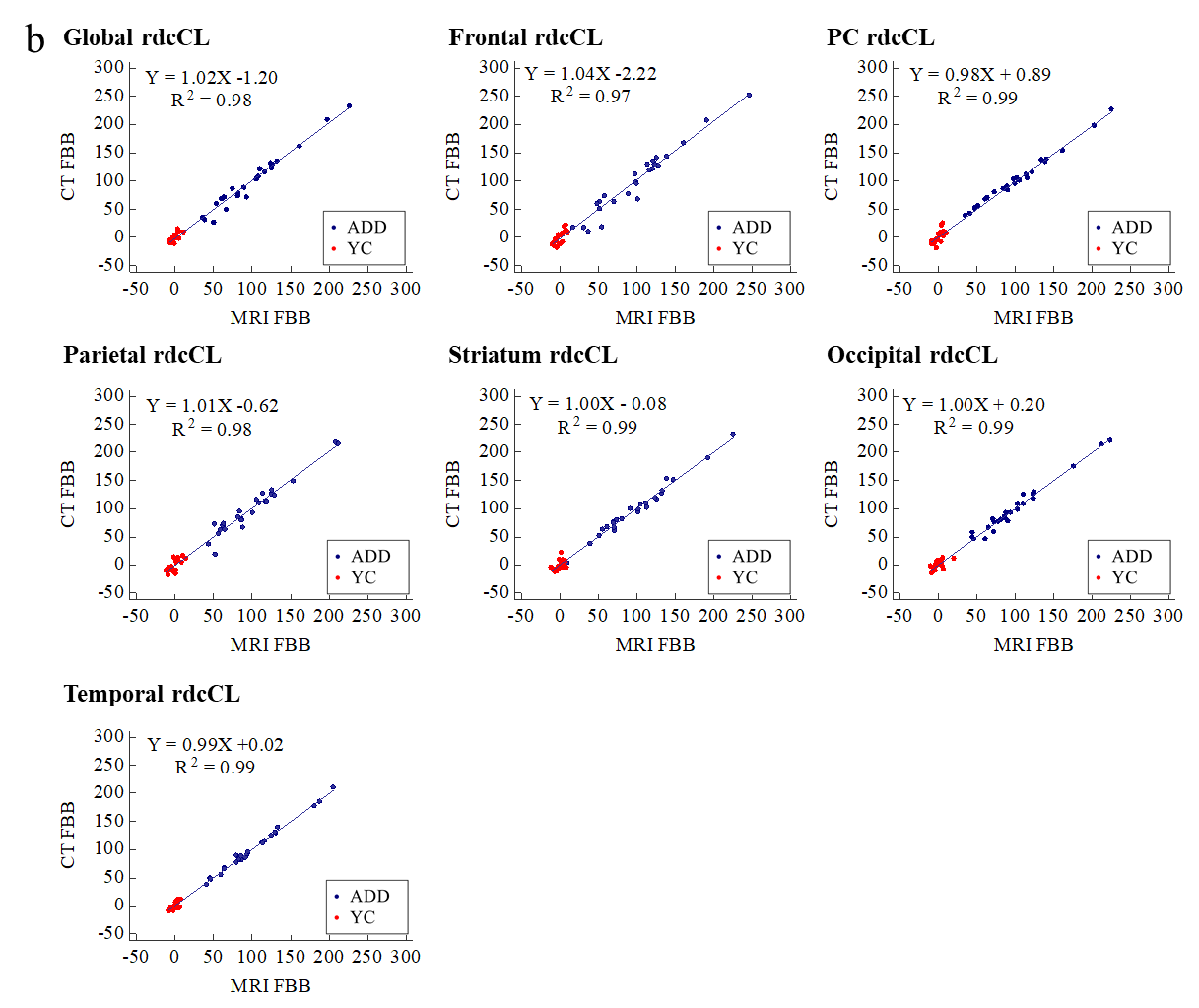

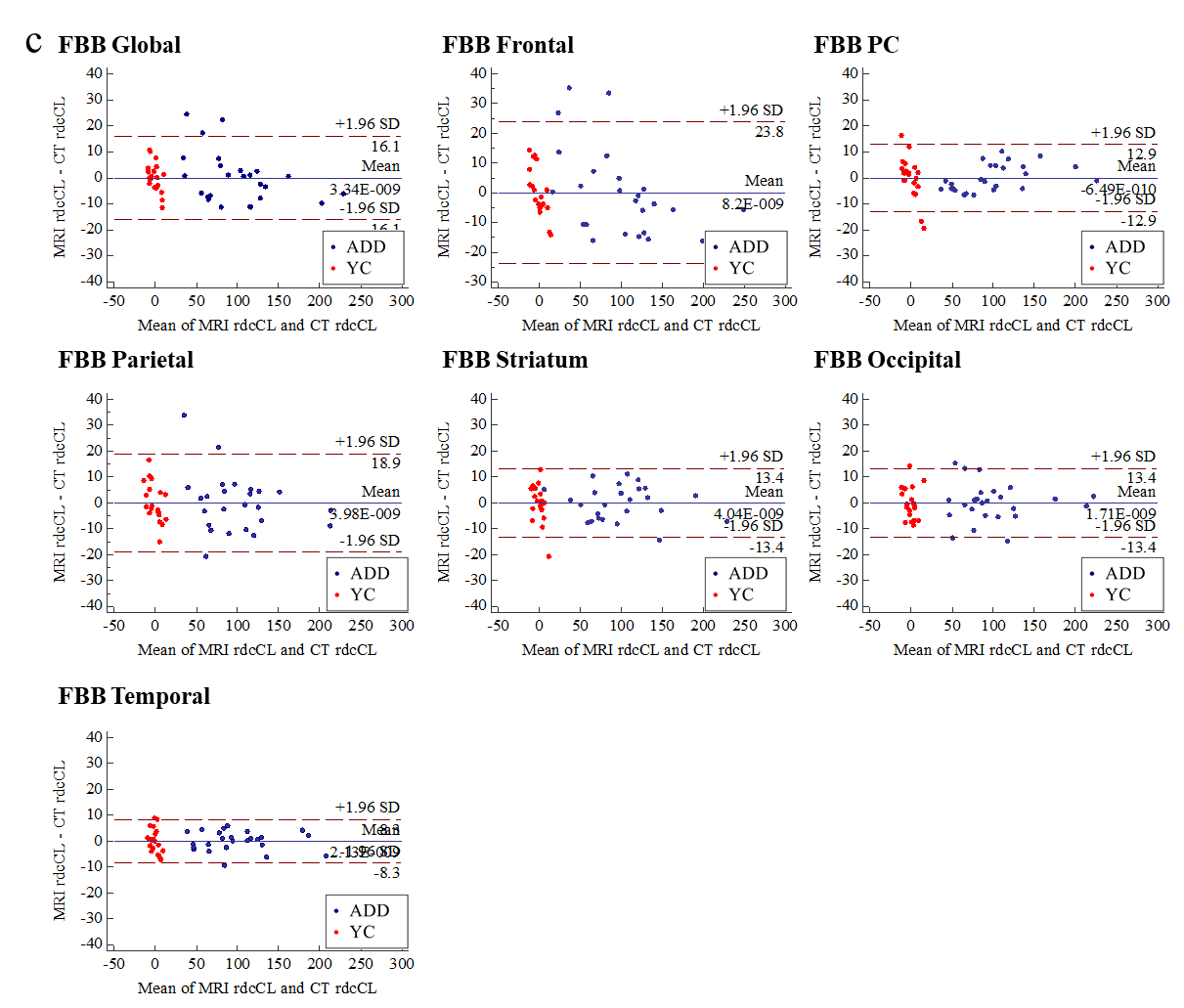


**Figure S6.** Correlation plots of **a** rdcSUVR and **b** rdcCL between rdcCL methods and Bland-Altman plots of rdcCL between **c** the methods for FBB PET.

Abbreviations: ADD, Alzheimer’s disease dementia; YC, younger control; FBB, ^18^F-florbetaben; rdcSUVR, standardized uptake value ratio derived from FMM-FBB CTX VOI and regional VOIs; rdcCL, Centiloid scales of FMM-FBB CTX VOI and regional VOIs; PC, posterior cingulate


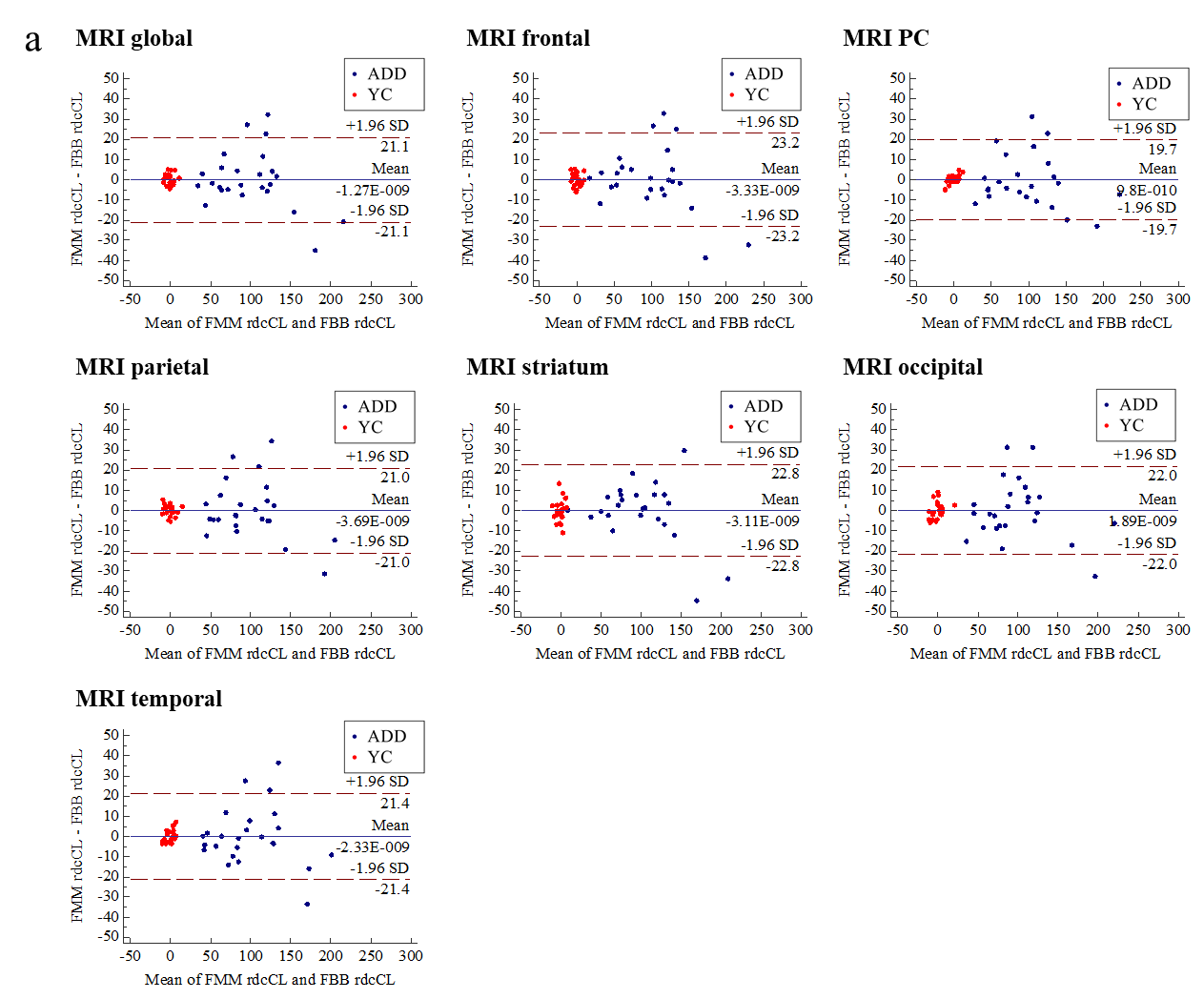

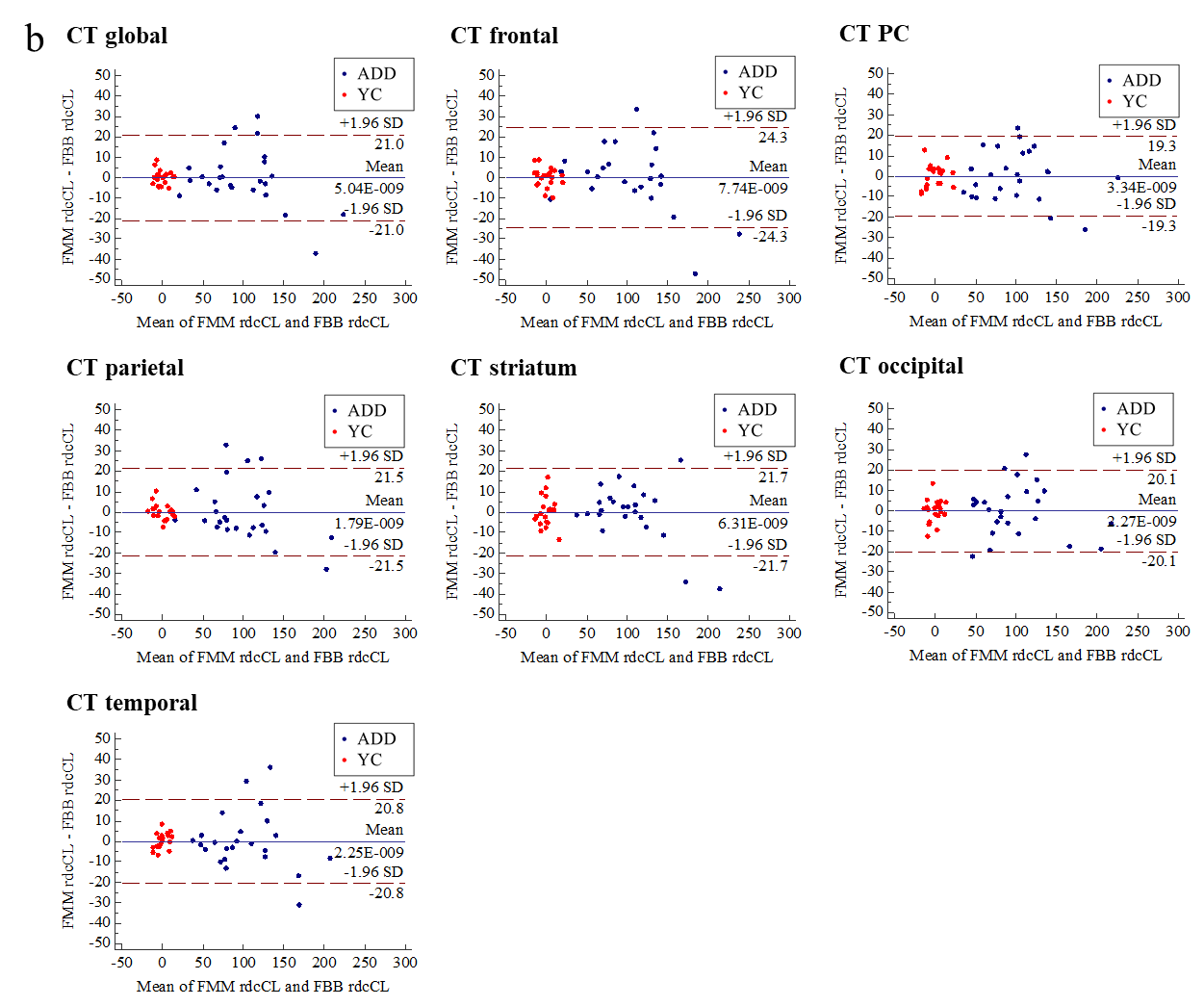


**Figure S7.** Plots of difference in rdcCLs between PET ligands for **a** MRI-based and **b** CT-based methods in the head-to-head cohort.

Abbreviations: ADD, Alzheimer’s disease dementia; YC, younger control; FMM, ^18^F-flutemetamol; FBB, ^18^F-florbetaben; rdcCL, Centiloid scales of FMM-FBB CTX VOI and regional VOIs; PC, posterior cingulate
